# A hidden gem in multidisciplinary antimicrobial stewardship: a systematic review on bedside nurses’ activities in daily practice regarding antibiotic use

**DOI:** 10.1093/jacamr/dlad123

**Published:** 2023-11-23

**Authors:** Maria Bos, Jeroen Schouten, Cindy De Bot, Hester Vermeulen, Marlies Hulscher

**Affiliations:** School of Social Work and Health, Avans University of Applied Sciences, ’s Hertogenbosch, The Netherlands; Scientific Center for Quality of Healthcare (IQ Healthcare), Radboud University Medical Center, Nijmegen, The Netherlands; Scientific Center for Quality of Healthcare (IQ Healthcare), Radboud University Medical Center, Nijmegen, The Netherlands; Department of Intensive Care Medicine, Radboud University Medical Center, Nijmegen, The Netherlands; School of Social Work and Health, Avans University of Applied Sciences, ’s Hertogenbosch, The Netherlands; Scientific Center for Quality of Healthcare (IQ Healthcare), Radboud University Medical Center, Nijmegen, The Netherlands; School of Health, HAN University of Applied Sciences, Nijmegen, The Netherlands; Scientific Center for Quality of Healthcare (IQ Healthcare), Radboud University Medical Center, Nijmegen, The Netherlands

## Abstract

**Background:**

Antimicrobial stewardship (AMS), the set of actions to ensure antibiotics are used appropriately, is increasingly targeted at all those involved in the antimicrobial pathway, including nurses. Several healthcare organizations have issued position statements on how bedside nurses can be involved in AMS. However, it remains unclear how nurses, in reality, contribute to appropriate antibiotic use.

**Objectives:**

To systematically search the literature to describe the activities bedside nurses perform regarding antibiotic use in daily clinical practice, in relation to the activities proposed by the aforementioned position statements.

**Methods:**

We searched MEDLINE, Embase, CINAHL and grey literature until March 2021. Studies were included if they described activities regarding antibiotic use performed by bedside nurses. Methodological rigour was assessed by applying the Mixed Method Appraisal Tool.

**Results:**

A total of 118 studies were included. The majority of the proposed nurses’ activities were found in daily practice, categorized into assessment of clinical status, collection of specimens, management of antimicrobial medication, prompting review and educating patient and relatives. Nurses may take the lead in these clinical processes and are communicators in all aspects of the antimicrobial pathway. Patient advocacy appears to be a strong driver of bedside nurses’ activities.

**Conclusions:**

Nurses’ activities are already integrated in the day-to-day nursing practice and are grounded in the essence of nursing, being a patient advocate and showing nursing leadership in safeguarding the antimicrobial treatment process. An essential element of the nursing role is communication with other stakeholders in the patient-centred antimicrobial pathway. Educating, engaging and empowering nurses in this already integrated role, could lead to a solid, impactful nursing contribution to AMS.

## Introduction

Antimicrobial resistance (AMR) is an increasing global threat.^1,2^ It is estimated that 33 110 patients die per year in the EU alone as a result of infections caused by resistant bacteria.^3^ One of the strategies to mitigate AMR is to use antibiotics in an optimal way and the set of actions to ensure this optimization is known as antimicrobial stewardship (AMS).^4^ Traditionally, AMS programmes are targeted on infectious disease specialists, medical microbiologists and pharmacists and only recently there has been an increasing focus on the role bedside nurses play with regard to antibiotic use.^5–13^

In 2015, Olans *et al*.^14^ used a modified Delphi process with 10 nurse educators to identify several areas in which nurses should be educated as a prerequisite for adequate participation in AMS programmes. These consensus-based activities were also used to further elaborate the potential contribution of nurses^6,15^ and became a solid foundation to the position papers of several international nursing and healthcare organizations.

**Figure 1.**
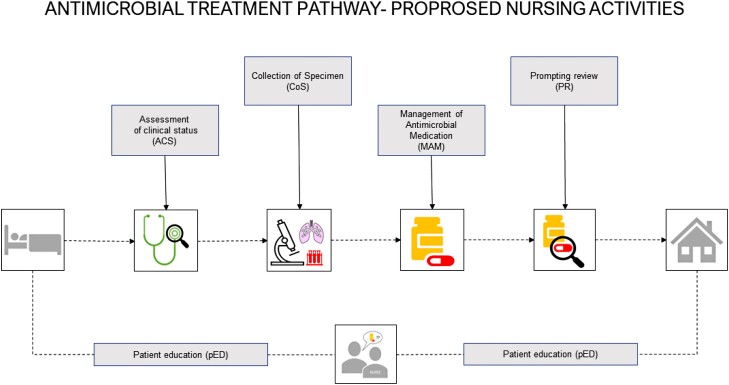
Antimicrobial treatment pathway—proposed nursing activities.

In 2017, the International Council of Nurses (ICN) published its revised Position Statement on the role of nurses in combatting AMR.^16^ Other healthcare organizations such as American Nurses Association (ANA), CDC, Australian Commission on Safety and Quality in Health Care (ACSQHC), European Federation of Nurses (EFN) and WHO followed,^7,17–19^ acknowledging nurses for the important role they can play in protecting the ‘power of antibiotic medication’.^16,20^ These position papers suggest that nurses may contribute by assessing and monitoring patients’ clinical status, participating in diagnostic culture and antimicrobial treatment management, and educate patients and families (Figure 1).^21,22^

However, it remains unclear how bedside nurses in their daily practice perform these proposed activities and how this relates to their role as described in the position papers.

This integrative review aims to describe the activities bedside nurses perform regarding antimicrobial use in daily practice.

## Methods

This integrative review (PROSPERO registration CRD42020161713) was conducted according to the methodology of Whittemore and Knafl, combining both quantitative and qualitative studies to create a holistic understanding of the phenomenon under study.^23^

We focused on nurses’ activities related to appropriate antimicrobial use suggested by the position papers/guidelines of professional healthcare and nursing organizations.^7,16–19,21,22^ These recommendations were summarized into five domains (Table 1). The review is reported according to the PRISMA guidelines.^24^

**Table 1.** Summary of bedside nurses’ activities with regard to (appropriate) antibiotic use (as proposed by guidelines and position papers)

Domain 1 Assessment of clinical status 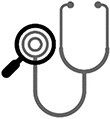	Assessment of clinical status Identify source of infection (assess, diagnose & identify appropriate precautions)^16,17,19^ Identify and escalate patients with signs of acute deterioration or infection^7^ Identify if patient has symptoms that justify diagnostics (e.g. does patient have symptoms that justify urine culturing?)^21^ Monitoring clinical status of the patient Monitor and report daily clinical status, including e.g. laboratory results (e.g. renal function test results)^17,19,22^ Monitor treatment outcomes^16^ Monitor capacity for oral intake^17,21^
Domain 2 Collection of specimen 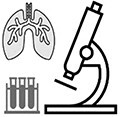	Collection of specimen Taking cultures before administering antibiotics^17,21,22^ Correctly taking cultures (avoiding contamination, unnecessary sampling/maintaining specimen quality)^7,19,21,22^ Communication and documentation of results Monitoring culture results (including sensitivity results?)^7,17^ Timely informing prescribers/physicians about results^17,22^
Domain 3 Management of antimicrobial medication 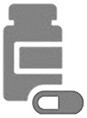	Allergy assessment Take and document allergy^17,19,21,22^ Administration of antimicrobial medication^16,17^ Medication reconciliation (including discharge)^7,18,22^ Timely administration (including first dose of antibiotics if patient has sepsis)^7^ Respect medication safety principles (5 ‘rights’ of medication administration)^7^ Reduce incidence of missed antimicrobial doses^7^ (Help) comply with surgical prophylaxis quality indicators^22^ Documentation of antimicrobials^7^ Document the indication & duration of antibiotic treatment ^7^ Documentation of administered antimicrobials^19^ Monitor adverse events, e.g. diarrhoea^7,17,19,22^
Domain 4 Prompting review 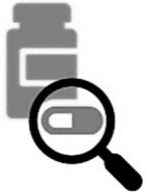	Prompting review of antimicrobial treatment^21^ Prompt prescribers to review antibiotic treatment (not further specified) Monitor treatment duration: need for continuation (Day 3 or/and Day 7)^22^ Prompt review of potential for IV-to-oral switch^17,21^ Arrange & coordinate follow-up for review of antibiotic treatment^7^ Prompt review of drug/bug mismatch, time-outs, antibiotic de-escalation^17,21^ Prompt assessment of suitability of patient for OPAT^22^ Prompt discussions on issues concerning antimicrobial therapy at the end of life with patients, carers and other members of the healthcare team as part of planning for end-of-life care^7^
Domain 5 Patient communication, education & information 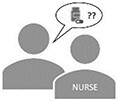	Educating patient and family/caregivers (including discharge information) Educate patient on appropriate use^18,21^ Inform patients on antibiotic timing, interaction with food, medication compliance^18,22^ Educate patients on adverse events^22^ Educate patients on when to ask for review if concerned^7^ Give information on which signs and symptoms to share with healthcare provider^21^ Give information on how to return leftover medication to pharmacy^22^ Encourage patient and peer professional interactions on antimicrobial prescription and therapy^22^

### Literature search

In March 2020 (and updated in March 2021), a systematic literature search was conducted in the following databases, from 2000 and onwards: PubMed/MEDLINE, Cumulative Index to Nursing & Allied Health Literature (CINAHL) and Excerpta Medica database (Embase). We developed search strings containing the keywords ‘antibiotic/antimicrobial’, ‘stewardship/plan/program/policy’ and ‘nurse’ or synonyms of these keywords ( File S1, available as Supplementary data at *JAC* Online). Reference lists of included articles were handsearched for additional studies, performing both forward and backward searches. Literature reviews were handsearched for additional eligible primary studies.

Since the topic of nurses’ involvement in antibiotic use is relatively young (first publication on this topic is dated 2011),^9^ we also performed an extensive grey literature search, using Web of Science, conference proceedings and websites of relevant professional organizations such as CDC, ESCMID, the Association for Professionals in Infection Control and Epidemiology (APIC) and The Society for Healthcare Epidemiology of America (SHEA), to identify additional unpublished studies.^25,26^ A simplified search string using keywords ‘antibiotic/antimicrobial’ and ‘nurse’ was used. Since conference abstracts are currently published in supplements of peer-reviewed journals and therefore were included in the updated search, we did not perform an additional search of the grey literature.

### Eligibility criteria

We included primary studies if they described activities regarding antibiotic use performed by bedside nurses in all healthcare settings. Bedside nurses were defined as nurses who perform direct patient care activities (Registered Nurses, Licensed Practical Nurses, Specialized Nurses, Nurse Specialists, and equivalents of those).^27,28^ Studies describing activities by nurse prescribers were excluded since prescribing antibiotics is not a regular activity of bedside nurses^29^ and these prescribing rights require additional registration.^30,31^ We made no restrictions regarding the methodology of the primary studies. Also, we included studies in both inpatient and outpatient settings, as well as settings in high-, low- and middle-income countries. We applied no language selection. If the original language was not fully mastered by the researchers, we translated the article by using an automated translating service.

For the grey literature search, we applied the same eligibility criteria. Additionally, if a conference abstract was subsequently published as a journal article, this article was included in the peer-reviewed literature screening process.^32^ If a conference abstract had insufficient data, the first author was contacted to request additional information to facilitate the inclusion decision.^32^

### Study selection/data extraction

Two researchers independently screened title and abstract of both peer-reviewed (M.H., M.B.) and grey literature results (J.S., M.B.), as well as the selected full-text articles. In case of disagreement on eligibility, discussion followed until consensus was reached. Any discrepancies between reviewers were resolved through consensus or consultation with a third reviewer. The screening and selection process was aided by Rayyan software.^33^ Data regarding study specifics and nurses’ activities were independently collected using Excel software by one researcher (M.B.), of which 50% was double-checked (J.H.). The following variables were collected: author, year of publication, aim of study, study design, characteristics of the participants, setting, country of study origin, nursing activities related to antibiotic use.

### Quality appraisal

The selected peer-reviewed literature was critically appraised by two researchers (M.B., C.B.) independently, using the Mixed Method Appraisal Tool (MMAT) by Hong *et al*.,^34^ which is applicable for different methodologies. However, methodological quality was not used as an exclusion criterium.

### Data analysis

All studies were uploaded in Atlas.ti 8 Windows software. Relevant text fragments were highlighted, coded and categorized into networks of comparable nurses’ activities. These activities were then compared with the proposed activities as described by the healthcare organizations (Table 1).

## Results

**Figure 2.**
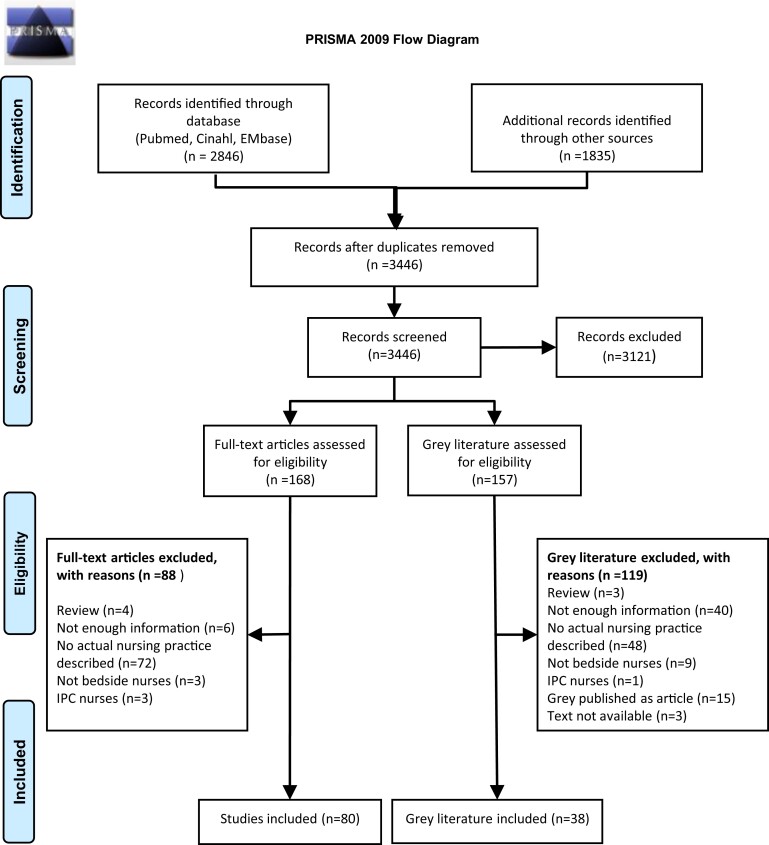
PRISMA 2009 flow diagram.

The search identified 3446 studies, of which the full text of 168 potentially relevant studies and 157 potential grey literature studies were assessed for eligibility. Altogether, 118 studies met the inclusion criteria [80 peer-reviewed and 38 grey literature (abstracts *n* = 35, thesis *n* = 3)] (see PRISMA flow diagram, Figure 2; and characteristics of included studies, Files S2 and S3).^10,35–151^

Most studies were hospital-based (*n* = 92; 78%)^10,35–37,39–44,46–48,51–56,60–66,68,69,71,72,74,75,78–85,87,89–97,99–104,106,108–111,115–117,119–127,129–131,133–136,138–146,148,149,151^ or described practices in long-term care facilities (LTCFs) and/or nursing homes (*n* = 20; 16.9%).^38,49,50,57–59,73,76,77,88,98,104,105,107,112–114,128,137,150^ The majority of the studies originated in the USA (*n* = 57; 48.3%)^37,38,47,48,50,53–55,61,66–68,74,76–82,86–88,90–92,95,98–101,104,108,109,112–117,121–124,126–129,132–134,137,141,142,145–147,150,151^ and Australia (*n* =17; 14.4%).^10,40–44,57–60,62,65,71–73,111,139^ Of the 118 studies, 43 (36.4%) focused primarily on bedside nurses’ activities.^10,38,40,45,47–49,57,58,61,66,68–72,77,78,80–83,85,89,92–94,100–102,104,106,110,121,125,134–137,139,147,149,151^ In the remaining 75 studies, information on nurses’ activities was extracted from studies addressing topics influencing antibiotic use, e.g. prescriber’s behaviour (*n* = 18; 15.3%).^35,36,42,44,57,60,65,73,84,88,90,96,99,106,107,112,114,131^

Of the selected studies, 60 (50.8%) reported on quality improvement^152^ projects.^36,37,44,46,50,53,55,57,60,61,63,64,67,68,76,78,79,82,86,87,89–91,95,99–103,108,109,113–117,119–124,127–129,131,133,136–146,149,150^

Of the peer-reviewed literature, 33 (41.3%) studies were classified as non-randomized studies,^10,36,37,46,50,53–55,57,61,64,67,68,75,76,78,79,82,86,87,89–91,95,99–103,108,109,113,114^ 26 (32.5%) were qualitative studies,^39–43,49,52,57,58,65,71–73,77,83,88,93,94,96–98,104–107,110^ 18 (22.5%) were quantitative descriptive studies such as surveys^35,38,45,47,48,51,56,62,66,69,70,74,80,81,84,92,111,112^ and three (3.8%) studies combined quantitative and qualitative designs.^44,60,85^

Upon critical appraisal, there was much variation in rigour of methodology. Thirty-six papers were defined as high quality, meeting ≥80% of the MMAT criteria,^39–43,49,52,55,57,58,62,65,71,73,77,78,80,81,83,84,86,88,90,92,93,95,96,98,104–108,110,112,114^ 18 papers were of moderate quality (40%–80% of the criteria were met)^10,36,38,44,45,50,51,54,57,66,67,69,74–76,78,94,99^ and 26 studies were of low quality (≤40% of the criteria were met).^35,37,46–48,53,56,60,61,64,68,70,72,82,83,87,89,91,97,100–103,109,111,113^ The most common flaw was a lack of information on the methodology (File S4).

## Main findings

Nurses’ activities are presented in relation to the summary of proposed activities (Table 2).

**Table 2.** Nurses’ activities in daily practice compared to as proposed by the healthcare organizations

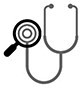	Domain 1 Assessment of clinical status	References
Assessment of clinical status (activity)	Identify source of infection (assess, diagnose & identify appropriate precautions)^16,17,19^	—
	Identify and escalate patients with signs of acute deterioration or infection^7^	^38,40,46,49,50,53,55,57–59,61,73,76,77,86,87,91–93,98,99,104,105,107,113,114,121,128,141,142^
	Identify if patient has symptoms that justify diagnostics (e.g. does patient have symptoms that justify urine culturing?)^21^	^39,50,57,61,77,104,105,107,113,115,122,128^
Monitoring clinical status of the patient	Monitor and report daily clinical status, including e.g. laboratory results (e.g. renal function test results)^17,19,22^	^50,88,93,107,110,128^
	Monitor treatment outcomes^16^	^40,49,93,103^
	Monitor capacity for oral intake^17,21^	—
Extra	Communication and/or documentation of signs and symptoms	^40,49,50,55,56,58,68,77,87,88,99,104,105,107,108,112–114,137,142,150^
	Assessment of clinical status as part of telephonic care to prevent treatment	^86^
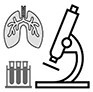	Domain 2 Collection of specimens	References
Collection of specimens	Take cultures before administering antibiotics^17,21,22^	^70,91,96,119^
	Take cultures correctly (avoiding contamination, unnecessary sampling/maintaining specimen quality)^7,19,21,22^	^39,46,50,57,61,63,66,75,77,91,93,96,99,104,108,113,115,120,122,124,127,133,146^
Communication and documentation of results	Monitoring culture results (including sensitivity results)^7,17^	^47–49,61,64,71,77,93,95,134,151^
	Timely informing prescribers/physicians about results^17,22^	^45,50,54,77,94,95,104,130,134^
Extra	Nurse initiate sample collection	^49,50,57,77,91,97,99,104,105,107,113^
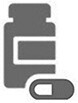	Domain 3 Management of antimicrobial medication	References
Assessment of antibiotic allergies	Take and document allergy for antibiotics^17,19,21,22^	^36,41,47,48,64,66,100,106,110,126,129,145^
Administration of antimicrobial medication	Administer antimicrobial medication^16,17^	^10,35–37,40–42,44,49,51–53,64,69,71,78–80,83–85,92,93,96,110,116,118,125,148^
	Perform medication reconciliation (including medication reconciliation at discharge of the patient)^7,18,22^	—
	Timely administration (including first dose of antibiotics if patient has sepsis)^7^	^40,41,45,53,65,71,79,83,85,91,92,94,96,102,119,121,138,143,144,149^
	Respect medication safety principles (5 ‘rights’ of medication administration)^7^	^40,41,45,49,53,56,66,67,70,81,83,85,93,94,106,110^
	Reduce incidence of missed antimicrobial doses^7^	^37,40,41,47,53,54,65,71,85,92,110^
	(Help) comply with surgical prophylaxis quality indicators^22^	^36,84,109,117,140^
Documentation of antimicrobials	Document the indication & duration of antibiotic treatment^7^	^93,131,139^
	Documentation of administered antimicrobials^19^	^35,64,83,93^
Monitor adverse events	Monitor adverse events, e.g. diarrhoea^7,17,19,22^	^50,62,74^
Extra	Nurse participates in treatment initiation	^40,45,49,73,77,88,90,105^
	Nurse participates in non-bedside AMS	^45,47,59,60,72,76,104,110^
	Nurse monitors therapeutic levels	^48^
	Nurse stops treatment based on culture results	^77^
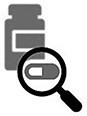	Domain 4 Prompting review	References
Prompting review	Prompt prescribers to review antibiotic treatment (not further specified)	^40,42,43,45,49,58,60,68,71,72,80,82,89,104,106,110,112,136^
	Monitor treatment duration: assess need for continuation on Day 3 or/and Day 7^22^	^40,44,45,48,59,80,89,92,93,101,103,123^
	Prompt review of potential for IV-to-oral switch^17,21^	^10,71,96^
	Arrange & coordinate follow-up for review of antibiotic treatment^7^	
	Prompt review of drug/bug mismatch, time-outs, antibiotic de-escalation^17,21^	^42,68,71,93,104,117^
	Prompt assessment of suitability of patient for OPAT^22^	^10,96^
	Prompt discussion on issues concerning antimicrobial therapy at the end of life with patients, family and members of the healthcare team as part of planning for end-of-life care^7^	^57,58^
Extra	Participation in AMS rounds (review)	^68,93,100,101,123^
	Prompt to prescribe	^40,43,49,58,73,98,105,107,112^
	Nurse discusses treatment based on results	^72,104^
	Nurse prompt prescriber to document indication & duration	^58,139^
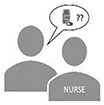	Domain 5 Patient communication, education and information	References
Educating patient and family/caregivers (including discharge information)	Educate patient on appropriate use^7,18^	^45,47,48,86,110,132^
	Inform patients on antibiotic timing, interaction with food, medication compliance^18,22^	^45,48,67,72,85,110,111,135,147^
	Educate patients on adverse events^22^	—
	Educate patient on when to ask for review if concerned^7^	—
	Give information on which signs and symptoms to share with healthcare provider^21^	^86^
	Give information on how to return leftover medication to pharmacy^22^	—
Encourage patient and peer professional interactions on antimicrobial prescription and therapy^22^		^58^
Extra	Nurse communicates culture results to patient & gives advice to stop antibiotics	^95^
	Nurse gives education about correctly taking cultures	^108^

### Domain 1—Bedside nurses’ assessment of clinical status

Overall, four of the six aspects of the first domain were addressed by studies describing how nurses assess the patient’s clinical status related to antibiotic use in daily practice.

The first aspect mentions that nurses should assess, diagnose and identify appropriate precautions to prevent transmission of microorganisms. Studies describing this activity in relation to AMS were not found.

The second aspect, identification and escalation of patients with signs of acute deterioration or infection, was the most frequent nursing activity (*n* = 30).^38,40,46,49,50,53,55,57–59,61,73,76,77,86,87,91–93,98,99,104,105,107,113,114,121,128,141,142^ Thirteen studies also described how nurses are in the lead by taking predetermined follow-up actions on their clinical assessment, guided by decision tools, algorithms and protocols,^49,50,53,55,61,77,87,91,99,121,128,141,142^ such as a neonatal sepsis calculator.^55,87,121,141,142^ Three studies mentioned that nurses, undertaking these actions, are advocating for the best care for their patients.^40,104,107^

Twelve studies described the third aspect, how nurses, in daily practice, identify whether patients have symptoms that justify diagnostics.^39,50,57,61,77,104,105,107,113,115,122,128^ For example, Zabarsky *et al*.^113^ described that nurses in an LTCF often initiate urine cultures, based on their clinical judgement.

The fourth aspect addresses that nurses monitor and report the clinical status of a patient. Six studies describe this activity in daily practice.^50,88,93,107,110,128^ Aspect five, how nurses monitor treatment outcomes in daily practice, was described by four studies.^40,49,93,103^ Nurses’ monitoring of oral intake capacity (aspect 6) was not found.

As an additional aspect of the proposed nurses’ activities in daily practice, communication and/or documentation of signs and symptoms by nurses, was found in 21 studies.^40,49,50,55,56,58,68,77,87,88,99,104,105,107,108,112–114,137,142,150^ Nurses, in daily practice, take the lead in communication in different ways, e.g. in an LTCF by recommending treatment based on signs and symptoms^49^ or communicating patients’ clinical status in nurse-driven AMS rounds.^68^

### Domain 2—Collection of specimens

The second domain targets how nurses contribute to AMS by collecting diagnostic specimens. All four aspects were described in daily practice.

The first aspect, nurses taking cultures before administering antibiotics, was described in four studies.^70,91,96,119^ One study described that this sampling process was nurse-led, as part of triage standing orders.^91^

The second aspect addresses that nurses should take cultures correctly. Nurses upheld this standard in 23 studies.^39,46,50,57,61,63,66,75,77,91,93,96,99,104,108,113,115,120,122,124,127,133,146^ Four of these studies also described that nurses initiated a discussion with the prescriber about the necessity of the cultures^61,115,122^ or initiated sampling.^113^ Three studies mentioned that, in daily practice, nurses communicate or document orders for cultures or document the specimen’s source.^50,93,108^

The third aspect concerns nurses’ monitoring of culture results. Eleven studies mentioned that this is daily nursing practice.^47–49,61,64,71,77,93,95,134,151^ One study reported that nurses led a culture follow-up process in paediatric patients, where, after physician review, nurses communicated negative culture results to the parents, together with the physician’s recommendation to discontinue antibiotics.^95^

The fourth aspect describes that nurses should timely inform the prescribers about the culture results. Nine studies mention this nursing communication in daily practice.^45,50,54,77,94,95,104,130,134^ Three studies reported that nurses go one step further, by appraising the results and, if deemed necessary, alarming the physician.^54,95,104^ One study reported that nurses advocate for patients’ best interests when communicating these results.^107^

In the summary of proposed nurses’ activities, there was no suggestion that nurses, in their daily process, should initiate sample collection for diagnostic purposes. However, nurses taking the lead in daily practice were described in 11 studies.^49,50,57,77,91,97,99,104,105,107,113^ In three studies this was considered a bad practice, especially related to UTI diagnostics.^77,104,105^

### Domain 3—Management of antimicrobial medication

This domain (Table 2) covers nurses’ activities related to the management of antimicrobial medication and includes 10 aspects, of which 9 were described in the literature.

The first aspect describes that nurses should take and document allergies for antibiotics, which was described as daily practice by 12 studies.^36,41,47,48,64,66,100,106,110,126,129,145^ Three of these reported that nurses take the lead in this, either by using a decision tool or algorithm when obtaining an allergy history^129,145^ or by participating in rounds with a hospitalist discussing antibiotic allergies.^100^

Administration of antimicrobial medication, the second aspect, was the second most frequently described nursing activity in daily practice (*n* = 29).^10,35–37,40–42,44,49,51–53,64,69,71,78–80,83–85,92,93,96,110,116,118,125,148^ Three studies also described that nurses, in relation to the medication administration process, perceive themselves as advocates for their patient, ensuring that the patient receives the best possible care.^40,41,83^ Communication about antimicrobial medication was seen as a vital element in daily practice, as was emphasized by Rout *et al*.^93^ in a study among ICU nurses.

The third aspect, medication reconciliation by nurses, was not covered in the studies.

Aspect four, timely administration of antibiotics by nurses, was described by 20 studies.^40,41,45,53,65,71,79,83,85,91,92,94,96,102,119,121,138,143,144,149^ Two studies reported on quality improvement projects where nurses take the lead by using triage standing orders to decrease time to antibiotics in neonates in the Emergency Department.^91,138^ Broom *et al*.^41^ described that nurses, fearing adverse clinical outcomes and driven by patient advocacy, timely administered antibiotics without pharmacist approval. In a study in an Australian Emergency Department, nurses reported that poor interprofessional communication negatively impacted the timely antibiotic administration.^65^

Regarding aspect five, nurses’ activities related to medication safety, the five Rs (‘the right patient, right drug, right dose, right route, and the right time’ protocol), were mentioned as a daily care activity in 16 studies.^40,41,45,49,53,56,66,67,70,81,83,85,93,94,106,110^

The sixth aspect concerns how nurses ensure that the incidence of missed antimicrobial doses is reduced. Eleven studies described that nurses contribute to this reduction in daily practice.^37,40,41,47,53,54,65,71,85,92,110^ Wong *et al*.^110^ reported that nurses take the lead in this by ‘smoothing’ the process of antibiotics, e.g. by verifying whether the pharmacy has been contacted. Three studies mentioned ‘brokering’ (ensuring the antibiotic is available) as nursing activity, which was driven by patient advocacy.^40,71,85^

The seventh aspect of the domain is how nurses (help) comply with surgical prophylaxis indicators, such as the correct timing of antibiotic administration before surgery. Five studies mentioned this nursing activity in daily practice.^36,84,109,117,140^ Baker *et al*.^117^ described how nurses lead this preoperative process, by using an algorithm to evaluate the correctness of the preoperative antibiotic.

The eighth domain aspect addresses how nurses, in daily practice, document the indication and duration of antimicrobial treatment. Three studies described this nursing activity in daily care,^93,131,139^ of which Lo *et al*.^139^ describe a nurse-led AMS intervention to encourage the medical team to document indication and planned treatment duration.

Documentation of administered antimicrobial medication by nurses in daily practice (aspect nine) was mentioned in four studies.^35,64,83,93^

The tenth aspect concerns nurses’ monitoring of adverse events of antimicrobial treatment. Three studies described this activity in daily practice.^50,62,74^

Four additional bedside activities were found. Nurses participate in treatment initiation, which may have different gradations. Eight studies described this nursing activity in daily practice,^40,45,49,73,77,88,90,105^ which ranges from selecting and starting antibiotics based on an algorithm,^77,88^ recommend treatment based on clinical assessment^49^ or give guidance to prescribers in what the nurse thinks is the best decision for this patient.^40^ One study mentioned that nurses may stop antibiotic treatment when the cultures are negative.^77^ Nurses also monitor therapeutic levels of the antibiotic treatment.^48^

Additional non-bedside AMS activities performed by nurses in daily practice were found in eight studies.^45,47,59,60,72,76,104,110^ Nursing participation included developing antimicrobial prescribing guidelines,^45,47^ ensuring implementation and use of protocols and guidelines, ^45,60,110^ promoting guideline adherence,^76,104^ teaching appropriate use^45,72^ and participating in audits.^45,59^

### Domain 4—Prompting review

Prompting, ‘encouraging or reminding someone of something’,^153^ contains seven aspects, all of which were found.

The first aspect of this domain, prompting prescribers to review antibiotic treatment, was described as nursing activity by 20 studies.^40,42,43,45,49,58,60,68,71,72,80,82,89,100,104–106,110,112,136^ Prompting may be an act of nursing leadership (described in 14 studies^40,42,44,49,58,60,68,71,72,80,82,106,110,136^) but was also considered an act of patient advocacy, striving for the best possible care.^40,58,60,71,72,104,110,136^

The second aspect describes that nurses can monitor treatment duration and assess the need for continuation on Day 3 and/or Day 7. Twelve studies described this daily practice,^40,44,45,48,59,80,89,92,93,101,103,123^ of which four studies emphasized the importance of this nursing role.^44,89,101,103^ According to one study, this is how they advocate for their patients.^40^

The third aspect, prompting review of the potential for IV-to-oral switch, was described by three studies.^10,71,96^ No study detailed the fourth aspect, where nurses arrange and coordinate follow-up for review of antibiotic treatment.

Prompting review of drug/bug mismatch, time-outs and antibiotic de-escalation (the fifth aspect) was described by six studies,^42,68,71,93,104,117^ where in five studies nurses took a leading role.^42,68,71,104,117^ Rout and Brysiewicz^93^ added that, by doing this, nurses are acting on behalf of their patient.

The sixth aspect, prompting assessment of the suitability of the patient for outpatient parenteral antibiotic therapy (OPAT), was described in two studies.^10,96^ The seventh aspect, prompting discussion about antibiotic therapy at the end of life, was described by two studies,^57,58^ where nurses see their role primarily as patient advocates and show leadership, especially in relation to AMS.

Four additional bedside nurses’ activities were described. Firstly, according to five studies, bedside nurses lead and participate in AMS rounds where antibiotic treatment is discussed in multidisciplinary cooperation.^68,93,100,101,123^

Secondly, nine studies reported that nurses prompt prescribers to start antibiotic treatment for their patients,^40,43,49,58,73,98,105,107,112^ thus showing leadership in daily practice (*n* = 4)^40,43,105,112^ or advocating for their patients (*n* = 2).^43,107^

As the third additional aspect, two studies mentioned that bedside nurses discussed treatment with the prescriber, based on the diagnostic results,^72,104^ although the prescribers were not always willing to listen.^104^

Lastly, two studies indicated that bedside nurses, in daily practice, led by prompting prescribers to document indication and duration of antibiotic treatment, thus advocating for their patients.^58,139^

### Domain 5—Patient communication, education and information

This domain contains seven aspects, of which five were described in daily practice.

The first aspect, patient and family education on appropriate use, was described in six studies^45,47,48,86,110,132^ as a nursing activity in daily practice.

The second aspect describes how nurses educate and inform patients on how to take their antibiotics (timing, interaction with food, medication compliance), which was reported as daily practice in nine studies.^45,48,67,72,85,110,111,135,147^

The education of patients on adverse events or when to ask for review when concerned (aspects three and four) were not found in the literature. Only one study mentioned the fourth aspect, that nurses give information on which signs and symptoms to share with healthcare providers.^86^ The fifth aspect, how nurses, in daily practice, educate patients on how to return leftover medication, was not addressed.

The sixth aspect, how nurses can encourage patient and peer professional interactions on antimicrobial prescription and therapy, was mentioned in one study.^58^

Additional activities of bedside nurses in this domain were found in two studies, where bedside nurses give patients education on how to correctly take cultures^108^ and where nurses communicate the negative culture results to the patient and family and give the physician’s advice to stop antibiotic treatment.^95^

## Discussion

This review aimed to describe the activities bedside nurses perform regarding antibiotic use in daily clinical practice, comparing them with the proposed nursing activities in the position statements by professional healthcare organizations (ICN, ANA, CDC, ACSQHC, EFN, WHO). We found that nurses, in daily practice, without question, already perform the majority of the proposed activities throughout the entire antimicrobial pathway.

These results nuance the findings of previous reviews, which stated that nurses remain disconnected from AMS activities^154^ or need improvement in communication skills regarding AMS,^155,156^ e.g. to question the prescriber.

Importantly, we also found three overarching intrinsic elements of these nurses’ activities that were not explicitly mentioned in the position papers. First, nurses’ activities appear to be grounded in patient advocacy, ‘striving for the best possible care for their patient’.^157^ Several attributes of patient advocacy,^158^ such as safeguarding the patient from errors, providing information about the patient’s diagnosis and treatment, and mediating (being a liaison between patient, family and healthcare workers), are clearly recognizable throughout nurses’ contribution to AMS, e.g. as nurses prompt review of the antimicrobial treatment.^40,58,60,71,72,104,110,136^ Patient advocacy may be a strong motivator for participation in AMS, which was also acknowledged in recent reviews by Gotterson *et al*.^154^ and van Huizen *et al.*^156^

Secondly, nurses, in daily practice, take the lead in many parts of the antibiotic treatment process, e.g. by initiating culture sampling^49,50,57,77,91,97,99,104,105,107,113^ or participating in AMS rounds.^68,93,100,101,123^ This clinical (often informal and hidden)^159^ bedside leadership may be an expression of the central position of nurses in patient care.^160^ The coordination of the healthcare trajectory^161^ is a contribution that is not always visible, recognized or valued.^162,163^ By uncovering this dimension of nursing leadership, we highlight a complementary building block for AMS in effective and safe patient care.^164,165^

Third, we found that communication (including documentation of information) is an important aspect of the nurses’ contribution^65,93,104,107,114^ and interwoven in all aspects of the antimicrobial pathway. In an overview article on the ‘past, present and future of nurses and AMS’, Olans *et al*.^166^ considered the nursing profession as a central hub in the AMS communication web, updating those who provide care as well as patients and their caregivers.

Appropriate antimicrobial use is considered a patient safety priority^167^ and part of providing high-quality patient care. Since effective interdisciplinary communication is essential for providing high-quality care,^168,169^ all members of the multidisciplinary healthcare team should continue to invest in effective communication throughout the antimicrobial pathway. To strengthen the team communication, barriers such as physician and nurse hierarchy^94,104,106^ or lack of knowledge and experience regarding antibiotic use^13,94,106,154,156,170–172^ should be addressed.

The findings of this review highlight that nurses’ contribution to AMS is already grounded in daily practice although nurses may not recognize their daily practice as contributing to AMS. Nurses’ actions are targeted at patients well-being and ensuring a smooth care trajectory, while not specifically aimed at reaching AMS goals. However, as a collateral benefit, these nursing goals align with the AMS goals of effectively treating infections, protecting patients from harm caused by unnecessary antibiotic use (e.g. toxic side effects, *Clostridioides difficile* infection) with the ultimate goal to prevent the development and spread of AMR.^21^

As a first step to enhance the nursing contribution to AMS programmes, an educational intervention can be applied. Education, as an implementation strategy, has been shown to improve AMS behaviour such as recognizing the appropriate response to treatment, communicating promptly when receiving laboratory results and collaborating with the interprofessional healthcare team to optimize antimicrobial treatment.^173^ As an example, Arnold *et al.*^174^ described an educational intervention aimed at nurses, to support them with clinical reasoning and communication process concerning suspected urinary tract infections (UTIs) in nursing home patients. This intervention importantly reduced the rate of antibiotic prescriptions and inappropriate treatment.

However, education (on its own) does not necessarily translate into change of practice.^175^ To fully live up to the stewardship role, nurses also need to apply leadership, to be committed to speaking up about issues related to the antimicrobial treatment. Olans *et al.*,^166^ in a summarizing paper, described that true multidisciplinary collaboration involving nurses has not yet been achieved and should be improved by emphasizing the collaborative character of AMS. Nurses need to ‘take a seat at the table’, while being acknowledged as a valuable and visible contributor. However, prior to establishing an effective partnership between prescribers and nurses, some practical issues should be addressed, such as knowledge gaps, time constraints and uncertainty about the nursing scope of AMS practice.^176^ Clarification of professional roles will increase mutual respect for the unique contribution of all those involved in AMS, which is essential for effective collaboration.^177^ Working in an environment where AMS is perceived as a collaborative effort and the responsibility of all those involved in the patient careway, is crucial for establishing a meaningful nursing contribution.^52,71^ Overall, nurses make up almost 50% of the global nursing and midwifery workforce^178^ and by involving nurses in AMS activities, the potential to achieve and consolidate the AMS goals may be unprecedented.^179^

To realize this potential and enable nurses to fully contribute to AMS, whether in high-, middle- or low-income countries, a tailored approach is needed to support nurses’ contribution.^8,180,181^ Global standards, guidelines and toolkits are already available for this purpose, e.g. the ‘WHO Competency Framework for Health Workers’ Education and Training on Antimicrobial Resistance’,^19^ the ‘Antimicrobial Stewardship Programmes in Health-Care Facilities in Low- and Middle-Income Countries: a WHO Practical Toolkit’,^182^ and the WHO’s ‘Health Workers’ Education and Training on AMR: Curricula Guide’.^183^

Future research, as advised by WHO in the Global Research Agenda on AMR, should investigate strategies to optimize antimicrobial therapy.^184^ Research focused on how to engage and sustain nurses’ contribution in AMS, as well as how to optimize the nurse–physician collaboration in AMS, can be seen as a very promising endeavour.

This review has several strengths. We performed an extensive and broad search and included grey literature, preventing the effects of publication bias.^185^ We included studies from all healthcare settings, which gives a broad understanding of nurses’ activities in both intramural and extramural care pathways showing the potential for further nursing engagement.

A limitation is that, due to the search strategy connected to AMS, studies with no apparent link to appropriate antibiotic use (e.g. studies that only describe nurses’ contribution to UTI diagnostics without highlighting AMS potential), were not included. Yet, we feel that due to the extent of our search results, this did not impact the identification of the most common activities of nurses in daily practice.

Secondly, as we focused in our review on the activities that bedside nurses perform with regard to appropriate antibiotic use, we did not address nurses with prescribing rights. These expert nurses form a special group of nurses who have undergone additional specialist training to acquire the competencies to prescribe medication. They are also bound by organizational and jurisdictional regulations.^30,31,186^ As recently detailed by Courtenay *et al*.,^187^ these nurse prescribers are not yet targeted in AMS programmes. Future research should also focus on this specific group of prescribers.

## Conclusions

Nurses’ activities, as proposed by international healthcare organizations, are already integrated into day-to-day nursing practice. These activities, throughout the entire antimicrobial treatment pathway, are grounded in the essence of nursing, being a patient advocate and showing leadership in safeguarding the antibiotic treatment process. An essential element of the nursing role is communication with other stakeholders in the patient-centred antimicrobial process. Educating, engaging and empowering nurses to embrace and acknowledge their contribution to AMS could lead to a solid, impactful contribution to safe healthcare across the globe.

## Supplementary Material

dlad123_Supplementary_Data

## Supplementary data

Files S1–S4 are available as Supplementary data at *JAC* Online.
