# Supplementary material for: A hidden gem in multidisciplinary antimicrobial stewardship: a systematic review on bedside nurses’ activities in daily practice regarding antibiotic use
Source: JAC Antimicrob Resist. 2023 Nov 23;5(6):dlad123. doi: 10.1093/jacamr/dlad123 (PMC10667038; doi:10.1093/jacamr/dlad123)
Supplement: dlad123_Supplementary_Data [file dlad123_supplementary_data.docx]

A hidden gem in multidisciplinary Antimicrobial Stewardship: a systematic review on bedside nurses’ activities in daily practice regarding antibiotic use

**SUPPLEMENTARY FILES S1-S4**

AUTHORS:

*Maria BOS ^1.2^

Dr Jeroen SCHOUTEN ^2.3^

Dr Cindy DE BOT ^1^

Professor Hester VERMEULEN ^2,4^

Professor Marlies HULSCHER ^2^

^1^ Avans University of Applied Sciences, ‘s Hertogenbosch, the Netherlands

^2^ Scientific Center for Quality of Healthcare (IQ Healthcare), Radboud University Medical Center, Nijmegen, the Netherlands

^3^ Department of Intensive Care Medicine, Radboud University Medical Center, Nijmegen, the Netherlands

^4^ School of Health, HAN University of Applied Sciences, Nijmegen, the Netherlands

Corresponding author:

Maria BOS

Scientific Center for Quality of Healthcare (IQ Healthcare)

Radboud University Medical Center

Kapittelweg 54

6525 EP Nijmegen, the Netherlands

Email: [rita.bos@radboudumc.nl](mailto:rita.bos@radboudumc.nl)

Tel.: 0031-6-42992204

Contents

[Supplementary file S1: Search Strings PubMed/Medline, CiNAHL, Embase 3](#_Toc148303208)

[Supplementary file S2 Characteristics of study (peer reviewed literature) 6](#_Toc148303209)

[Supplementary file S3 Characteristics of included studies (grey literature) 53](#_Toc148303210)

[Supplementary file S4: Critical appraisal with Mixed Method Appraisal Tool (MMAT) 72](#_Toc148303211)

# Supplementary file S1: Search Strings PubMed/Medline, CiNAHL, Embase

**PubMed**

(“Anti-Infective Agents”[Mesh] OR antimicrobial[tiab] OR anti-microbial[tiab] OR anti-biotic*[tiab] OR antibiotic*[tiab])

AND

(plans OR program OR programs OR plan OR policy OR policies OR use OR "Antimicrobial Stewardship"[Mesh])

AND

("Nurses"[Mesh] OR "Nursing Staff"[Mesh]) OR "Licensed Practical Nurses"[Mesh] )

--------------------------------------------------

("Nurses"[Mesh] OR "Nursing Staff"[Mesh] OR "Allied Health Personnel"[Mesh] OR "Nurse's Role"[Mesh] OR nursing[tiab] OR nurse[tiab] OR nurses[tiab])

AND

(Antimicrobial plan*[tiab] OR Antimicrobial program*[tiab] OR Antimicrobial polic*[tiab] OR Antimicrobial use[tiab] OR Antimicrobial steward*[tiab] OR Anti-microbial plan*[tiab] OR Anti-microbial program*[tiab] OR Anti-microbial polic*[tiab] OR Anti-microbial use[tiab] OR Anti-microbial steward*[tiab] OR Antibiotic plan*[tiab] OR Antibiotic program* [tiab] OR Antibiotic polic*[tiab] OR Antibiotic use[tiab] OR Antibiotic steward*[tiab] OR Anti-biotic plan*[tiab] OR Anti-biotic program* [tiab] OR Anti-biotic polic*[tiab] OR Anti-biotic use[tiab] OR Anti-biotic steward*[tiab] OR "Antimicrobial Stewardship"[Mesh])

------------------------------------------------------------

**Embase**

(exp nurse/ OR Nursing Staff/ OR health practitioner/ or nursing assistant/ OR exp nursing care/ OR nursing.ti,ab,kw. OR nurse.ti,ab,kw. OR nurses.ti,ab,kw.)

AND

(Antimicrobial plan*.ti,ab,kw. OR Antimicrobial program*.ti,ab,kw. OR Antimicrobial polic*.ti,ab,kw. OR “Antimicrobial use”.ti,ab,kw. OR Antimicrobial steward*.ti,ab,kw. OR Anti-microbial plan*.ti,ab,kw. OR Anti-microbial program*.ti,ab,kw. OR Anti-microbial polic*.ti,ab,kw. OR “Anti-microbial use”.ti,ab,kw. OR Anti-microbial steward*.ti,ab,kw. OR Antibiotic plan*.ti,ab,kw. OR Antibiotic program* .ti,ab,kw. OR Antibiotic polic*.ti,ab,kw. OR Antibiotic use.ti,ab,kw. OR Antibiotic steward*.ti,ab,kw. OR Anti-biotic plan*.ti,ab,kw. OR Anti-biotic program* .ti,ab,kw. OR Anti-biotic polic*.ti,ab,kw. OR Anti-biotic use.ti,ab,kw. OR Anti-biotic steward*.ti,ab,kw. OR Antimicrobial Stewardship/)

-------------------------------------------------------------

**CINAHL**

((MH "Nurses+") OR (MH "Nursing Assistants") OR (MH "Nurses by Educational Level+") OR (MH "Nurses by Role+") OR (MH "Nurses by Specialty+") OR (MH "Nurses, Other+") OR (MH "Nursing Role") OR (MH "Physician Assistants") OR (MH "Community Health Workers") OR (MH "Nursing Care+") OR TI(nursing OR nurse OR nurses) OR AB(nursing OR nurse OR nurses))

AND

TI (Antimicrobial plan* OR Antimicrobial program* OR Antimicrobial polic* OR Antimicrobial use OR Antimicrobial steward* OR Anti-microbial plan* OR Anti-microbial program* OR Anti-microbial polic* OR Anti-microbial use OR Anti-microbial steward* OR Antibiotic plan* OR Antibiotic program* OR Antibiotic polic* OR Antibiotic use OR Antibiotic steward* OR Anti-biotic plan* OR Anti-biotic program* OR Anti-biotic polic* OR Anti-biotic use OR Anti-biotic steward*) OR AB(Antimicrobial plan* OR Antimicrobial program* OR Antimicrobial polic* OR Antimicrobial use OR Antimicrobial steward* OR Anti-microbial plan* OR Anti-microbial program* OR Anti-microbial polic* OR Anti-microbial use OR Anti-microbial steward* OR Antibiotic plan* OR Antibiotic program* OR Antibiotic polic* OR Antibiotic use OR Antibiotic steward* OR Anti-biotic plan* OR Anti-biotic program* OR Anti-biotic polic* OR Anti-biotic use OR Anti-biotic steward* ) OR (MH "Antimicrobial Stewardship")

------------------------------------------------------------------

Addition for Embase:

(Antimicrobial plan*.ti,ab,kw. OR Antimicrobial program*.ti,ab,kw. OR Antimicrobial polic*.ti,ab,kw. OR "Antimicrobial use".ti,ab,kw. OR Antimicrobial steward*.ti,ab,kw. OR Anti-microbial plan*.ti,ab,kw. OR Anti-microbial program*.ti,ab,kw. OR Anti-microbial polic*.ti,ab,kw. OR "Anti-microbial use".ti,ab,kw. OR Anti-microbial steward*.ti,ab,kw. OR Antibiotic plan*.ti,ab,kw. OR Antibiotic program* .ti,ab,kw. OR Antibiotic polic*.ti,ab,kw. OR "Antibiotic use".ti,ab,kw. OR Antibiotic steward*.ti,ab,kw. OR Anti-biotic plan*.ti,ab,kw. OR Anti-biotic program* .ti,ab,kw. OR Anti-biotic polic*.ti,ab,kw. OR "Anti-biotic use".ti,ab,kw. OR Anti-biotic steward*.ti,ab,kw. OR Antimicrobial Stewardship/)

# Supplementary file S2 Characteristics of study (peer reviewed literature)

| **AUTHOR(S)** | **AIM** | **DESIGN** | **PARTICIPANTS** | **HEALTHCARE SETTING** | **COUNTRY** | **NURSING DOMAIN** | **DESIGN MMAT** | **MMAT-SCORE** |
| --- | --- | --- | --- | --- | --- | --- | --- | --- |
| Abahamye (2016) ^35^ | To establish whether antibiotic prescribing at Mseleni hospital is done according to the above principles of rational antimicrobial prescribing to establish the factors that influence the choice and outcomes of antimicrobial therapy at Mseleni hospital. | Prescription audit | Prescribers at wards and at outpatient department; 100 prescriptions (60 inpatient-40 outpatient) to all in- and outpatients of the Mseleni hospital, to whom antibiotics were prescribed | Hospital (district ) | South Africa | Antimicrobial medication management | Quantitative descriptive studies | 0 |
| Aiken (2013) ^36^ | Developing and implementing a surgical antibiotic prophylaxis (ap) policy as an intervention to change healthcare practitioners’ prescribing behaviour in a government hospital in Kenya. | Quality improvement study | 100 patients undergoing surgical procedures involving overnight admission | Government hospital | Kenya | Antimicrobial medication management | Non-randomized studies | 60 |
| Almaki (2017) ^37^ | To investigate the barriers to timely antibiotic administration in septic surgical intensive care unit (sicu) patients, and based on the barriers, create and implement a multidisciplinary bundle to decrease the prescription to antibiotic administration time. | Pre- and postintervention study (quality improvement study) | Adult patients, admitted to surgical intensive care units (sicu) who received vasopressants and broad-spectrum antibiotics for septic shock (pre-intervention n=29/post-intervention n=33)  survey: nurses (n=63) | Hospital | USA | Antimicrobial medication management  Prompting review | Non-randomized studies | 40 |
| Beeber (2021) ^38^ | To examine what RNs consider to be the most important resident characteristics and clinical information when they make decisions about whether to notify a clinician about suspected UTIs in NH residents. | Survey | Registered nurses practicing in a nursing home in the USA (n=881) | Nursing home | USA | Assessment of clinical status | Quantitative descriptive studies | 60 |
| Black (2019) ^39^ | To learn about health care providers’ perceptions of current antimicrobial use and stewardship, including barriers and facilitators to improving antimicrobial use at acute care hospitals in nova scotia. | Qualitative research study (9 focus groups and 3 semi- structured interviews | 24 pharmacists, pharmacy students, 14 physicians, 16 nurses and nurse practitioners from 5 hospitals | Hospital | Canada | Assessment of clinical status  Collection of specimen | Qualitative studies | 100 |
| Broom, Broom, Kirby & Scrambler (2017) ^40^ | To broaden the social analysis of antibiotic decisions, to reveal the potential role and influence of nurses therein. | Qualitative study (semi-structured interviews) | Nurses (n=30) who work in departments regularly involved in infection management | Hospital | Australia | Assessment of clinical status  Antimicrobial 00.  medication management  Prompting review | Qualitative studies | 100 |
| Broom, Broom & Kirby (2019) ^43^ | To systematically examine the influences on antimicrobial use across a broad range of hospital settings, to provide clarity around potential barriers to effective and sustained optimization of use. | Qualitative (semi-structured interviews) | Doctors (n=85), nurses (n=79), pharmacists (n=31), managers (n=27) in 5 hospitals | Hospital | Australia | Prompting review | Qualitative studies | 100 |
| Broom, Broom, Kirby, Gibson & Post  (2017) ^40^ | To explore the experiences of hospital doctors and nurses regarding antibiotic use, with a focus on respiratory clinicians’ perceptions of AMS interventions in a hospital setting. | Qualitative (semi-structured interviews) | Respiratory doctors (n=13) and nurses (n=15) in 2 hospitals | Teaching hospital | Australia | Antimicrobial medication management | Qualitative studies | 100 |
| Broom, Tee, Broom, Kelly, Scott & Grieve  (2019) ^44^ | To report the antibiotic prescribing patterns before and after the intervention and a qualitative analysis of the experience of the intervention. | 1.pre- and post-intervention audit by retrospective chart review 2.qualitative assessment of the intervention | 1.all adults (>18 years of age) admitted under general surgery with complicated intra-abdominal infections, who had received a definitive surgical source control procedure were enrolled.(pre-intervention n=23, post-intervention n=22) 2. Nurses (n=4), doctors (n=12), pharmacists (n=2) | Hospital | Australia | Antimicrobial medication management  Prompting review | Mixed methods studies | 75 |
| Broom, Kirby, Gibson & Post  (2017) ^41^ | To explore the perspectives of pulmonary clinicians on antibiotic use in hospital pulmonary infections. | Qualitative study (semi-structured interviews) | Pulmonary doctors and nurses (n=28) | Teaching hospital | Australia | Antimicrobial medication management  Prompting review | Qualitative study | 100 |
| Bulabula (2018) ^45^ | To assess the current involvement of nurses in the use and management of antimicrobials and their training in antimicrobial stewardship (AMS) across Africa. | Online questionnaire | Nurses (n=173) | Not defined | Africa (multiple countries) | Collection of specimen  Antimicrobial medication management  Prompting review  Patient communication, education and information | Quantitative descriptive studies (non-comparative study (survey)) | 60 |
| Bunsow (2015) ^46^ | To determine the capacity of hcps in the identification of sepsis and the impact of a telephone call from a specialist in clinical microbiology (cm) in the early recognition of sepsis. | Prospective clinical trial study | Patients who had blood cultures drawn (intervention n=150/control n=150) | Teaching hospital | Spain | Assessment of clinical status  Collection of specimens | Non-randomized studies | 20 |
| Cadavid (2017) ^47^ | To capture bedside RN roles and to determine the antimicrobial related education and training hospitals provide them. | Online survey | Nurse education directors (n=19) or nurse education designees (clinical nurse specialist/bedside nurse educator (n=9), director of nursing/chief nursing officer (n=4), nurse administrators (n=2) from 34 hospitals | Acute care hospital | USA | Collection of Specimen  Antimicrobial Medication Management  Collection of specimens  Patient communication, education and information | Quantitative descriptive studies | 20 |
| Carter (2019^48^ | To describe the perceived:  1. leadership support for nurses' involvement in antibiotic stewardship  2. clinical nurses' knowledge and training in antibiotic stewardship 3. clinical nurses' role and responsibilities in antibiotic stewardship. | Online survey | Infection preventionists (n=207) | Hospital | USA | Collection of Specimens  Antimicrobial Medication Management  Prompting review  Patient communication, education and information | Quantitative descriptive studies | 0 |
| Chaaban (2019) ^49^ | To investigate the nurse’s role in antibiotic prescribing decisions for the treatment of suspected infections in Nursing Home residents, as well as to understand how the inter-professional collaboration between nursing staff and prescribing doctors is developed in such context. | Qualitative study design based on an ethnographic approach (observations and semi-structured interviews) | Nurses (coordinating nurses n=4, registered nurses responsible for implementation of drug prescription orders, n=12), doctors (coordinating n=3, prescribing n=2), working in 5 nursing homes | Nursing home | France | Assessment of clinical status  Collection of specimens  Antimicrobial Medication Management  Prompting review | Qualitative studies | 100 |
| Cooper (2017) ^50}^ | 1. To address the insufficient assessment of LTC residents with suspected UTI by implementing an evidence-based, multifaceted program 2. to reduce inaccurate UTI diagnoses and inappropriate antibiotic treatment by improving clinicians’ knowledge of ASB and assessment of UTI | Quality improvement study | Period 1: average census of 100 residents period 2; average census of 83 residents  frontline nursing staff: no number of characteristics described | LTC/rehabilitation facility | USA | Assessment of clinical status  Collection of specimens  Antimicrobial Medication Management | Non-randomized studies | 60 |
| Cooper (2018) ^51}^ | To quantify frequency, volume and dose of drug discarded within administration sets in the clinical setting. | Non-interventional observational feasibility study | Intravenous administration sets of 6 clinical areas with high intravenous infusion (oncology day ward, 2 surgical wards, cardiac ICU, cardiac high-dependency unit, emergency admissions unit) | Hospital | Great Britain | Antimicrobial Medication Management | Quantitative descriptive studies | 60 |
| Currie (2020) ^52^ | To explain the mechanisms influencing implementation of a national programme for AMS in acute-care hospitals across Scotland, using NPT as an interpretive framework to explore multiprofessional perspectives. | Exploratory qualitative study | Implementation lead clinicians (n=27, qualitative interviews) frontline practitioners (doctors, pharmacists, nurses, n=72/focus groups) | Hospital | Great Britain | Antimicrobial Medication Management | Qualitative studies | 100 |
| Daniels (2018) ^53^ | To decrease the mean time to administration of antibiotics in patients admitted to the BMT unit with first fever to <60 minutes. | Before-after study (quality improvement study) | Bone marrow transplant unit patients with developed a first fever (n=650) | Hospital | USA | Assessment of clinical status  Antimicrobial Medication Management | Non-randomized studies | 0 |
| Davis (2016^54^ | To determine the value of a pharmacist driven antimicrobial stewardship service in the ED | Retrospective chart review | Period 1: 499 patients with positive culture, of which 42 eligible for follow-up period 2: 473 patients with positive cultures, of which 30 eligible for follow-up | Tertiary care hospital non-trauma emergency department | USA | Collection of specimens  Antimicrobial Medication Management | Non-randomized study | 60 |
| Dhudasia (2018) ^55^ | To describe the implementation of the SRC in obstetric and newborn care practice and quantify the proportion of infants born at 36 weeks’ gestation who were administered empirical antibiotics and/or subjected to laboratory testing for risk of EOS before and after the use of the SRC-based approach | Retrospective cohort study | Infants born at ≥36 0/7 weeks’ gestation pre-intervention n=5692 post-intervention n=6090 | Teaching hospital (university health care system/academic perinatal center) | USA | Assessment of clinical status | Non-randomized studies | 100 |
| Dos Santos (2016) ^56^ | To identify risk factors in the management of antimicrobial drugs by the nursing team | Retrospective descriptive document-based analysis | Patients from cancer or haematological clinics, using antimicrobial drugs, with nursing information between 2008 and 2011 | Hospital | Brazil | Assessment of clinical status  Antimicrobial medication management | Quantitative descriptive studies | 0 |
| Dowson, Friedman, Marshall, Stuart, Buising, Rajkhowas, Gotterson & Kong(2020) ^59^ | To explore how health professionals perceive antimicrobial use and potential AMS activities near the end of life in ACH's | Qualitative (one-to-one semi-structured interviews) | Twelve nurses (2 enrolled nurses, 10 RNs), 5 GPs, 2 pharmacists, and 1 GP registrar (the Australian equivalent of a fellow) mean years professional experience: 21 (range 4 months-42 years) | ACH (aged care home) | Australia | Assessment of clinical status | Qualitative studies | 100 |
| Dowson, Friedman, Marshall, Stuart, Busing, Rajkhowa, Gotterson &Kong (2020) ^58^ | To describe the perspective of health professionals on antimicrobial use near the end of life in aged-care home and investigate the potential opportunities for nurses to undertake antimicrobial stewardship activities near the end of life in aged-care homes | Qualitative (one-to-one semi-structured interviews) | Twelve nurses (2 enrolled nurses, 10 RNs), 5 GPs, 2 pharmacists, and 1 GP registrar (the Australian equivalent of a fellow) | ACH(aged care home) | Australia | Assessment of clinical status  Patient communication, education and information | Qualitative studies | 100 |
| Dowson, Marshall, Buising, Friedman, Kong & Stuart (2019) ^57^ | To determine the feasibility of implementing nurse-initiated PCR testing of respiratory specimens in nursing home settings and to compare antibiotic prescribing prior to and during implementation. | Pragmatic, historically controlled study. | Nursing homes (n=3) | Nursing home | Australia | Assessment of clinical status  Collection of specimens | Non-randomized studies | 60 |
| Ervin (2020) ^60^ | To gather information about opinions and practices of antimicrobial prescribing in order to generate an effective intervention aimed at increasing compliance with the therapeutic guidelines for antibiotic use at 3 health services with inconsistencies in antimicrobial audit results | Mixed-method design (semi-structured interview/pre-determined response questions) | Medical practitioners (n=7) senior nurses (n=13) | Hospital | Australia | Antimicrobial Medication Management  Prompting review | Mixed methods | 0 |
| Fabre (2020) ^61^ | To evaluate the impact of a nurse- driven URcx stewardship intervention among adult inpatients with or without indwelling urinary catheters in a 24- bed general medicine unit at a large academic center. | Quasi-experimental study (before-after) | Intervention-unit: patients at adult medicine unit (before n=118, after n=107) Control unit: patient at similar adult medicine unit (before n=148, after n=195) | Hospital | USA | Assessment of clinical status  Collection of specimens | Non-randomized studies | 40 |
| Fehily (2015) ^62^ | To identify the extent of healthcare workers (HCW) awareness of their patients’ adverse drug reaction, and antibiotic use in hospital | Cross-sectional survey | Doctors, pharmacists, nurses (n=183) (derived: nurses n=60) | Tertiary referral hospital | Australia | Antimicrobial Medication Management | Quantitative descriptive studies | 100 |
| Geerlinks (2020) ^64^ | To use lean six sigma methodology, a quality improvement initiative, to improve time to antibiotics (TTA) for children with chemotherapy-induced febrile neutropenia presenting to the emergency department | Prospective cohort study with historical comparison | Pediatric oncology patients (n=26) with fever, presenting at emergency department of tertiary care hospital | Hospital | Canada | Collection of Specimen  Antimicrobial Medication Management | Non-randomized studies | 40 |
| Gillespie (2013) ^10^ | To assess the influence of nurse education on antibiotic use and clinical practice. | Interviews (face-to-face/open-ended questionnaire) | 79 nurses (48 hospital Monash/31 Dendenong) from 6 wards at 2 campuses | Hospital tertiary referral health service | Australia | Antimicrobial Medication Management  Prompting review | Quantitative descriptive studies | 60 |
| Goulopoulos (2019^65^ | To explore the attitudes and beliefs of Australian ED clinicians towards antimicrobial stewardship in the ED. | Qualitative research study (semi-structured, one-to-one interviews) | ED doctors (n=8), nurses (n=8), pharmacists (n=6) and hospital administrators (n=0?) Working in Australian public hospital ed's | Hospital (secondary & tertiary referral center) | Australia | Antimicrobial Medication Management | Qualitative studies | 100 |
| Greendyke (2018) ^66^ | To explore their knowledge, attitudes, and practices in antimicrobial stewardship activities and to identify opportunities for additional nursing involvement in ASP’s. | Survey | Nurses working in inpatient setting or emergency department (n=451) | Academic hospital (5 campuses) | USA | Collection of specimen  Antimicrobial Medication Management | Quantitative descriptive studies | 60 |
| Grimes-Holsinger (2002) ^67^ | To evaluate the efficacy of a skills checklist, which included a standardized instruction sheet based in part on Rutledge and Donaldson’s recommendations for reducing the amount of time required to make a patient independent in the administration of an antibiotic. | Quasi-experimental | Patients receiving antibiotic therapy in the home (n=105)  Nurses who provided care were employed by the infusion therapy company | Home care | USA | Patient communication, education and information | Non-randomized studies | 60 |
| Ha (2019) ^68^ | To describe the clinical outcomes of a bedside nurse driven AMS intervention in a community regional medical center. | Before-after study | Staff and patients at a 31-bed medical step-down telemetry unit (number not specified) | Hospital | USA | Assessment of clinical status  Prompting review | Non-randomized studies | 0 |
| Hoefel (2014) ^69^ | To study vancomycin nursing administration by nurses. | Prospective observational study, | 18 participants doing 47 procedures | University hospital | Brazil | Antimicrobial medication management | Quantitative descriptive studies | 60 |
| Jayaweerasingham (2019) ^70^ | Identifying the level of knowledge, beliefs and practices of nursing students at a government nurses training school in Sri Lanka. | Descriptive cross-sectional study | 199 nursing students (all started clinical training) | Not defined | Sri Lanka | Collection of specimens  Antimicrobial medication management | Quantitative descriptive studies | 20 |
| Kilpatrick (2019) ^72^ | To explore and to describe nurses’ understanding, perception, and knowledge of their role in infection prevention and control (IPC) and AMS when providing care for children with AD | Qualitative exploratory descriptive study (focus group & semi-structured interviews ) | Registered nurses (n=16), working in emergency department, short-stay medical ward and dermatology outpatient clinic | Tertiary metropolitan children's hospital | Australia | Antimicrobial Medication Management  Prompting review  Patient communication, education and information | Qualitative studies | 20 |
| Kirby (2020) ^71^ | Explore Australian hospital nurses’ views on antimicrobial resistance and antimicrobial stewardship (AMS) in a hospital setting, in order to better understand the opportunities for and challenges to integration of nursing staff in antimicrobial optimization within hospital settings | Qualitative design (semi-structured interviews) | Nurses (n=86) in 4 different hospital settings (remote (<100 beds), regional, tertiary capital city (<500 beds) and regional private hospital (<200 beds) | Hospital | Australia | Collection of specimen  Antimicrobial medication management  Prompting review | Qualitative studies | 100 |
| Le (2006) ^74^ | To evaluate  1. the incidence and common types of adverse drug reactions among hospitalized children  2. the frequency of adverse drug reaction reporting by health care providers  3.the follow-up processes resulting from adverse drug reactions | Retrospective cohort study | Pediatric patients who experienced an adverse drug reaction between January 1, 1995, and December 31, 2004 | Community-based tertiary care children teaching hospital | USA | Antimicrobial Medication Management | Quantitative descriptive studies | 60 |
| Lim (2014) ^73^ | To explore the attitudes and perceptions of key healthcare providers towards AMS interventions in Australian residential aged care facilities (RACFs) | Qualitative study (semi-structured interviews and focus groups) | Nursing staff (n=40 (4 executive nurses, 15 nurse-unit manager, 21 registered nurses), 15 general practitioners, 6 pharmacists from 12 RACFs | RACF | Australia | Assessment of clinical status  Antimicrobial medication management  Prompting review | Qualitative studies | 100 |
| Lin (2012) ^75^ | To reduce BCC rates in the emergency department by use of an educational intervention and one-on-one feedback | Quality improvement   (before-after study} | Clinical nursing personnel at the emergency department (n=unknown) | Medical centre (hospital ) | Taiwan | Assessment of clinical status  Antimicrobial medication management  Prompting review | Non-randomized studies | 60 |
| Linnebur (2011) ^76^ | To evaluate the impact of a multidisciplinary intervention that included academic detailing on adherence to national nursing home–acquired pneumonia (NHAP) guidelines related to use of antibiotics. | Mixed-methods, quasi-experimental, unblinded | 16 nursing homes (8 intervention, 8 control) | Nursing home | USA | Assessment of clinical status  Antimicrobial medication management | Non-randomized studies | 60 |
| Lohfeld (2007) ^77^ | To examine the views of nursing staff and administrators in Long Term Care Facilities (LTCFs) regarding a clinical pathway for managing urinary tract infections UTIs) in Long Term Care Facility (LTCF) residents | Exploratory single case study | 19 individual interviews with administrators and 10 focus group interviews with nurses (n=52), 23 registered nurses (RN), 22 registered practical nurses (RPN), 4 licensed practical nurses (LPN), 4 bachelor of science nursing degree) from long-term care facilities(8 Canadian, 2 USA) | LTCF | USA/Canada | Assessment of clinical status  Collection of specimen  Antimicrobial medication management  Prompting review | Qualitative studies | 100 |
| Mclaughlin (2016) ^78^ | To assess the impact of a practice improvement project that used a multidisciplinary educational intervention to improve nursing adherence to use of secondary tubing and SPT to administer antibiotics | Practice improvement project (before-after survey + compliance audit) | Emergency nurses (n=151 before, n=60 after) | Hospital (Level II, academic emergency department) | USA | Antimicrobial medication management | Non-randomized studies | 80 |
| Mclaughlin (2017) ^79^ | 1. To determine whether the implementation of an IVP cephalosporin antibiotic protocol would improve time from provider order to administration for all ED patients who were ordered a cephalosporin antibiotic 2. To determine whether the intervention would lower costs and improve ED nursing satisfaction. | Before-after study | Pre-intervention: emergency department patients who received an i.v. cephalosporin by infusion over 30 min (n=1146) post-intervention: all emergency department patients who received the antibiotic by IVP over 2-5 min (n=1110) | Hospital | USA | Antimicrobial medication management | Non-randomized studies | 60 |
| Merrill (2019) ^80^ | To describe the nurse’s self-reported knowledge of antimicrobial use and resistance and gauge their attitude toward involvement in as efforts | Descriptive survey | 316 bedside nurses from 3 hospital | Hospital | USA | Antimicrobial medication management  Prompting review | Quantitative descriptive studies | 80 |
| Monsees (2020) ^82^ | 1.To implement a nurse-driven antibiotic engagement tool (AET) that addresses 4 critical activities in antibiotic optimization:  a. clarifying antimicrobial indication  b. examining duration  c. discontinuing therapy  d. converting from i.v. to p.o. therapy.  secondary aim was 2. to evaluate the effectiveness the ADIOS AET on nurses’ confidence, satisfaction, and understanding of antibiotic plans of care on 4 participating units to evaluate the feasibility of the implementation. | Quality improvement study; pre-post intervention design (survey) | Nurse (n=121), (pre-intervention n=71, post-intervention n=50)) from 3 not-for-profit hospitals | Hospital | USA | Prompting review | Non-randomized studies | 40 |
| Monsees (2018) ^81^ | To identify nurses’ roles and confidence in engaging in stewardship practices by conducting a survey of pediatric staff nurses employed at a 354-bed freestanding children’s hospital with a well-established prospective  audit and feedback stewardship program. | Online survey | Staff nurses in pediatric hospital (n=180) | Paediatric academic centre | USA | Antimicrobial medication management | Quantitative descriptive studies | 80 |
| Mula (2019) ^83^ | To examine workaround behaviors that nurses and doctors employ to address the challenges encountered during their antibiotic stewardship efforts at a tertiary hospital in Malawi. | Qualitative case study (focus groups, participant observations, interviews) | Pharmacists and laboratory technologists (n=8), senior (n=6) and junior (n=6) medical doctors, nurse (n=79, observations), nurses (n=13, interviews) from male and female ward | Tertiary hospital | Malawi | Antimicrobial medication management | Qualitative studies | 100 |
| Mula (2019) ^85^ | To explore nurses’ levels of adherence to the ‘five rights’ of antibiotic administration and factors influencing their practices | Sequential mixed method (prospective observational case study followed by interviews) | 49 observations of patients diagnosed with pneumonia receiving antibiotics nurses of male and female wards (n=23) | Tertiary hospital | Malawi | Antimicrobial medication management | Mixed methods | 40 |
| Musmar (2014) ^84^ | To find the pattern of antimicrobial prophylaxis use by evaluating time of the first dose, antibiotic selection and duration after surgery in three governmental hospitals in Northwest Bank/Palestine during 2011. | Observational prospective cohort study | Patients with abdominal, orthopedic and gynecological operations (n=400) in 3 hospitals | Government hospital | Palestine | Antimicrobial medication management | Non-randomized studies | 100 |
| Perez (2020) ^87^ | To reduce, in the first 6 months, the percentage of sepsis workups in infants born 35 weeks by 40% and the exposure of antibiotics in the same group of infants by 30%. | Quality improvement study | NICU(not defined) in community hospital | Hospital | USA | Assessment of clinical status | Non-randomized studies | 40 |
| Pittenger (2015) ^86^ | To examine the effectiveness and cost impact of a newly developed ARI care pathway | Retrospective cohort study with time series analysis. | 52,266 patients seeking care for ARI in 7 primary care clinics | Primary care clinics | USA | Assessment of clinical status  Patient communication, education and information | Non-randomized studies | 100 |
| Ramly (2020) ^88^ | To identify barriers to reducing antibiotic overuse and strategies to address them to improve antibiotic prescribing. | Qualitative descriptive study | Nursing home professionals (n=68, leadership, nurses and prescribers) from 6 nursing homes) | Nursing home | USA | Assessment of clinical status  Antimicrobial medication management | Qualitative studies | 100 |
| Raybardhan (2020) ^89^ | To develop and implement a non-ASP-led prescriber prompt for assessing duration of antimicrobial use in the critical care unit. | Quality improvement study ITS (intermittent time series) | Critical care nurses (no further characteristics provided) | Community hospital | Canada | Prompting review | Non-randomized studies | 20 |
| Recabal (2020) ^90^ | To evaluate the impact of implementing a new standardized algorithm to select antibiotic prophylaxis prior to prostate needle biopsies (PNB) on the rate of infectious complications after PNB | Observational cohort study | Men who underwent PNB between Jan1, 2011-Jan 31, 2012 (a) (n=584 )and Jan 1, 2014 and Jan 31, 2015 (b, n=654) | Hospital (tertiary care, outpatient department) | USA | Antimicrobial medication management | Non-randomized studies | 80 |
| Reynolds (2020) ^91^ | 1. To increase the percentage of neonates aged 0 days to 28 days undergoing an evaluation for SBI in the Emergency Department who receive antibiotics within 120 minutes of arrival from baseline 19% to 80% by June 30, 018, and sustain this for at least 6 months. 2. To decrease time in minutes from ED arrival to antibiotic administration in the target population and increasing TSO use for eligible infants. | Quality improvement study | Infants aged 0-28 days, suspected of serious bacterial infection, undergoing evaluation at pediatric emergency department (n=1162) | Hospital | USA | Assessment of clinical status  Collection of specimen  Antimicrobial medication management | Non-randomized studies | 20 |
| Roberts (2017) ^92^ | To evaluate the knowledge, practices and perceptions of critical care nurses regarding antibiotic initiation in patients with newly recognized septic shock. | Survey | Critical nurses (n=100) at 320-bed academic institution | Hospital | USA | Assessment of clinical status  Antimicrobial medication management | Quantitative descriptive studies | 80 |
| Rout (2017) ^93^ | To explore AMS team members’ perceptions of the role of the ICU nurse within the AMS team. | Qualitative design (semi-structured interviews) | Nursing (n=8) and non-nursing (n=7) members of the ICU team | Hospital | South Africa | Assessment of clinical status  Collection of specimen  Antimicrobial medication management  Prompting review | Qualitative studies | 80 |
| Rout (2020) ^94^ | To explore the views of healthcare professionals regarding barriers to the antimicrobial stewardship role of the nurse in intensive care in a private hospital in Kwazulu-Natal, South Africa. | Qualitative descriptive study | Nurses (hospital management (n=2), clinical ICU nurses (n=6), microbiologist (n=1), anesthetists (n=2), physician (n=2), surgeons (n=2)) | Hospital | South Africa | Collection of specimen  Antimicrobial medication management | Qualitative studies | 60 |
| Saha  (2017) ^95^ | To develop and implement a protocol for follow-up management of negative urine culture results to reduce inappropriate antibiotic exposure in the NCH off-campus UC network. | Quality improvement study | Pediatric patients receiving empiric antibiotic therapy for a UTI and subsequent negative urine culture result (n=910) | 6 UC (urgent care) centres of a tertiary care academic hospital | USA | Collection of specimen  Patient communication, education and information | Non-randomized studies | 80 |
| Schmitz (2013) ^97^ | To assess the current practice in BC testing in ICUs and labs across four European countries. | Qualitative (semi-structured telephone interviews) | ICU directors, ICU residents, ICU nurses, lab directors, and lab managers of 79 microbiological laboratories (labs) and 59 intensive care units | Hospital/laboratory | France, Germany, Italy, United Kingdom | Collection of specimens | Qualitative studies | 20 |
| Schouten (2007) ^96^ | To understand the barriers to optimal performance with respect to six key recommendations of antibiotic treatment for community acquired pneumonia (CAP) | Qualitative (semi-structured interviews & focus groups) | 9 residents, 6 consultants, 2 microbiologists, 1 clinical pharmacist (interviews ), 2 nurses Emergency Department, 2 nurses pulmonology ward, 2 residents (group interviews ) | Secondary care hospital | Netherlands | Collection of specimen  Antimicrobial medication management  Prompting review | Qualitative studies | 100 |
| Seshadri (2020) ^98^ | To describe nursing home staff experiences and perceptions of the factors that impact the sustainability of an antibiotic stewardship program (ASP). | Qualitative descriptive study | Senior leaders (n=12) "other staff ( nurse practitioners and physician assistants, assistant directors of nursing, pharmacist, infection preventionists, nurse managers (RNs), licensed practical nurses and certified nursing assistants)(n=36) in 9 nursing homes | Nursing home | USA | Assessment of clinical status  Prompting review | Qualitative studies | 100 |
| Settelmeyer (2018) ^99^ | To evaluate the use of an evidence-based protocol for patients presenting to the emergency department with throat pain to determine the effect on the number of patients who left without being seen (LWBS), patient length of stay, and appropriate antibiotic prescribing. | Quality improvement study | Patients with throat pain, presenting at Emergency Department | Community hospital | USA | Assessment of clinical status  Collection of specimen | Non-randomized studies | 60 |
| Shukla (2020^101^ | To reduce antibiotic usage in the NICU (quality improvement project) | Quality improvement study; pre-post intervention design | Level III NICU in academic medical center | Hospital | USA | Antimicrobial medication management  Prompting review | Non-randomized studies | 20 |
| Sloan (2020) ^100^ | To develop and implement an evidence-based, nurse-led interprofessional AMR to improve assessment and management of antimicrobial therapy in an adult acute-care medical center, and to measure its effect on broad-spectrum antimicrobial use, duration of treatment, and de-escalation of therapy. | Quality improvement study; pre-post intervention design | Adults over age 18 on the inpatient hospitalist service with length of stay over 48 hours, with a prescribed intravenous antimicrobial, in 4 medical-surgical units in a regional medical center | Hospital | USA | Antimicrobial medication management  Prompting review | Non-randomized studies | 40 |
| Sutton (2017) ^102^ | To describe a nurse-initiated quality improvement (QI) project that improved the care of critically ill patients in a New Zealand tertiary ICU. | Quality improvement study | 18 bed general ICU in tertiary level hospital (not further specified) | Hospital | New Zealand | Antimicrobial medication management | Non-randomized studies | 0 |
| Tanner (2020^103^ | To evaluate the impact of our ambulatory p-OPAT service | Before-after study | All children initiated on iv antibiotics for non-complex infections likely to be primarily managed in local as well as regional hospitals, managed in the ambulatory p-OPAT in regional children's hospital (before n=67, after n=78) | Hospital | Great Britain | Assessment of clinical status  Prompting review | Non-randomized studies | 40 |
| Valmadrid (2021) ^104^ | To enhance understanding of how nursing communication and relationship dynamics affect how Long Term Care Facilities (LTCF) residents are treated in the Emergency Department, and identify barriers to optimal antibiotic stewardship throughout a frequent transition of care (LTCF to the ED and back) in this high-risk population | Qualitative design (semi-structured interviews) | Staff members of LTCFs and EDs (n=32) (LTCF nurses n=12, LTCF medical directors n=4, ED nurses n=6, ED physicians n=10) | LTCF/hospital | USA | Assessment of clinical status  Collection of specimen  Antimicrobial medication management  Prompting review | Qualitative studies | 100 |
| Van Buul (2014^105^ | To examine factors that influence antibiotic prescribing in Long Term Care Facilities (LTCF), and present a conceptual model that integrates these factors | Qualitative design (semi-structured interviews) | 13 physicians  13 nursing staff (4 RN, 4 LPN, 4 nursing assistants), from nursing home (n=10) and residential care (n=3) | LTCF | Netherlands | Assessment of clinical status  Collection of Specimen  Antimicrobial medication management  Prompting review | Qualitative studies | 100 |
| Van Gulik (2020) ^106^ | The aim of this study was to explore organizational leaders’ and clinical nurses’ perceptions of the strengths and weaknesses of the current AMS, clinical governance structure and activities at a tertiary referral center in Thailand. | Case-study design (semi-structured interviews /focus group discussion) | Interviews: organizational leaders (n=15), focus groups (n=3): infection control nurses (n=7), senior nurses (n=5), junior nurses (n=6) (total n=18) | Hospital | Thailand | Antimicrobial medication management  Prompting review | Qualitative studies | 100 |
| Walker (2000) ^107^ | To explore the perceptions, attitudes and opinions of physicians and nurses involved in the process of prescribing antibiotics for asymptomatic bacteriuria in institutionalized elderly people. | Qualitative design (focus groups) | Physicians (n=22) and nurses (n=16) providing care to residents of Long Term Care Facilities (LTCF) | LTCF (community based) | Canada | Assessment of clinical status  Collection of specimen  Antimicrobial medication management  Prompting review | Qualitative studies | 80 |
| Walters (2019) ^108^ | 1.To increase the percentage of targeted patients with suspected UTI having appropriately ordered and collected specimens to 100% 2.To increase the proportion of targeted patients receiving algorithm- recommended antibiotic at discharge to 80%. | Before-after study (quality improvement) | Children aged 3 months-12 years ,who were evaluated for suspected uncomplicated UTI in the pediatric ED and discharged. (n=458) | Hospital (academic medical center with pediatric ED) | USA | Assessment of clinical status  Collection of specimen | Non-randomized studies | 80 |
| Webb (2006^109^ | To describe the interventions to address the problem of patients not receiving appropriate preoperative antibiotics in a timely fashion | Before-after study (quality improvement) | Surgical patients (n=not defined) | Hospital | USA | Administration of antimicrobial medication | Non-randomized studies | 20 |
| Wong (2020) ^110^ | To understand nurses’ perceptions of their roles in antibiotic stewardship and to further elucidate the facilitators and barriers that influence their empowerment and active involvement in antibiotic stewardship in hospitals. | Exploratory qualitative study | Registered nurses (n=104) of 3 tertiary public hospitals | Hospital | Singapore | Assessment of clinical status  Antimicrobial medication management  Prompting review  Patient communication, education and information | Qualitative studies | 100 |
| Yeoh  (2020) ^111^ | To assess whether patients were receiving adequate information about their antimicrobial treatment, who was delivering this information, and how patients would like this information to be delivered. | Survey | Patients (n=54) of geriatric (n=26), general medicine (n=13), rehabilitation (n=9) and infectious disease wards (n=6) | Hospital | Australia | Patient communication, education and information | Quantitative descriptive studies | 20 |
| Yogo  (2016) ^112^ | To describe the epidemiology, clinical characteristics, diagnostic evaluation, and antibiotic prescribing patterns for skin infections in nursing homes to identify opportunities to improve antibiotic utilization. | Retrospective cohort study | 12 nursing home (total no. of patients 985) yielding 100 eligible patients with skin infections | Nursing home | USA | Assessment of clinical status  Prompting review | Non-randomized studies | 80 |
| Zabarsky (2008) ^113^ | To reduce inappropriate treatment of ASB | Prospective study | Long Term Care Facilities (LTCF), (nursing staff, n=80),  (prescribers n=6) | LTCF | USA | Assessment of clinical status  Collection of specimen | Non-randomized studies | 40 |
| Zimmerman (2014) ^114^ | To determine whether antibiotic prescribing can be reduced in nursing homes using a quality improvement (QI) program that involves providers, staff, residents, and families. | Quasi-experimental trial of quality improvement program | 12 nursing homes (6 comparison, 6 intervention) | Nursing home | USA | Assessment of clinical status | Non-randomized studies | 80 |

# Supplementary file S3 Characteristics of included studies (grey literature)

| **AUTHOR** | **CONFERENCE**  **or**  **THESIS** | **AIM** | **DESIGN** | **NUMBER OF PARTICIPANTS +CHARACTERISTICS** | **HEALTH-CARE SETTING** | **COUNTRY** | **TOPIC** | **NURSING DOMAIN** |
| --- | --- | --- | --- | --- | --- | --- | --- | --- |
| Adams (2020) ^115^ | Decennial 2020: International Conference On Healthcare Associated Infections | Not formally described: to reduce the frequent ordering of urine cultures for conditions and symptoms not supported by current IDSA guidelines | Quality improvement study | Physicians, nursing staff | Hospital | USA | Reduction of urine diagnostics | Assessment of clinical status  Collection of specimens |
| Averre (2018) ^116^ | Doctorate of Nursing Practice | To improve the administration process of piperacillin/tazobactam on one nursing unit | Quality improvement study | Nurses (n=73) | Hospital | USA | Antibiotic administration | Antimicrobial Medication Management |
| Baker (2014) ^117^ | American Society Of Perianesthesia Nurses Annual Conference | Will a new prospective review program by the clinical staff in the PAES and pre-operative departments lead to an increased level of compliance with core measures? | Quality improvement study | Nurses, pharmacist, leadership PAES & peri-operative department (n=not specified) | Hospital | USA | Perioperative ab prophylaxis | Antimicrobial Medication Management  Prompting review |
| Beausir (2013) ^118^ | ESCP International Workshop. Improving Patient Care Through Collaborative Practice | To assess cost effectiveness of anti-infective agents by pump administrations with hospital-based home care compared to a conventional management care (HBHC / community nurses)? (edited) | Retrospective cohort study | 36 patients treated with anti-infective agents | Home care | United Kingdom | OPAT | Antimicrobial Medication Management |
| Beukes (2014) ^119^ | 16th International Congress on Infectious Diseases | To improve antibiotic hangtime in bone marrow transplant unit | Quality improvement before-after study | Bone marrow transplant unit | Hospital | South Africa | Improvement of antibiotic hang-time | Collection of specimens  Antimicrobial Medication Management |
| Bowling (2010) ^120^ | Hospital Medicine 2010, April 8-11, Washington, D.C., USA | To reduce our blood culture contamination rates from 4.4% on a single general medicine ward to a rate of less than 2%, | Quality improvement before-after study | Nursing and laboratory staff, hospitalist faculty | Hospital | USA | Improvement of blood culture contamination | Collection of specimens |
| Bridges (2016) ^121^ | American College of Emergency Physicians Research Forum | The goal of this study was to determine whether utilization of a nurse-initiated sepsis protocol would identify emergency department patients at risk of sepsis and improve compliance with the 3-hour sepsis bundle. | Quality improvement study (before-after study) | Patients with diagnosed with sepsis in emergency department (before) n=296 Patient suspected or diagnosed with sepsis in the emergency department (after) n=91 | Hospital | USA | Sepsis | Assessment of clinical status  Antimicrobial Medication Management |
| Campbell (2016) ^122^ | ID Week | Not formally described in abstract (derived: to prevent unnecessary antibiotic use with regard to UTI) | Quality improvement study (before-after study) | ICU patients (not specified) | Hospital | USA | Reduction of inappropriate ab use in UTI/ASB | Assessment of clinical status  Collection of specimens |
| Castillo (2019) ^123^ | ID Week | To evaluate the impact of interdisciplinary rounds as a strategy to optimize antimicrobial use (AU) in the community hospital setting. | Quality improvement before-after study | Hospitalists, clinical pharmacists, case managers, nurses | Hospital | USA | Effect of multi-disciplinary rounds on antibiotic use | Prompting review |
| Choi (2019) ^124^ | American Thoracic Society Conference | No formal aim stated (derived: report on the reduction of blood culture contamination) | Quality improvement study | 17,757 blood cultures drawn | Hospital | USA | Reduction of blood culture contamination | Collection of specimens |
| Cockbum (2016) ^125^ | FIS/HIS | To evaluate the role of the AMT nurse in achieving improved antimicrobial stewardship related to gentamicin administration and prescribing | Before-after study | Nursing staff of 18 medical and surgical wards (n=unknown) | Hospital | United Kingdom | Antibiotic administration | Antimicrobial Medication Management |
| Dahan (2019) ^126^ | American Academy of Allergy, Asthma & Immunology (AAAAI) Annual Meeting | We hypothesized that the recording of drug allergies in the paediatric population is also imprecise and sought to assess the steps involved in recording drug allergies in a paediatric inpatient unit. | Prospective chart review | Paediatric inpatient unit [n=145] | Hospital | USA | Optimizing approaches to drug allergies | Antimicrobial Medication Management |
| Dockery (2010) ^127^ | Blood And Marrow Transplantation Meetings | To implement several initiatives which will enhance awareness of infection prevention in our sensitive immunocompromised patient population resulting in a decrease in the amount of central line infections and false positive blood cultures drawn on the bone marrow transplant unit at UAB hospital. | Protocol for quality improvement | BMT staff | Hospital | USA | Quality improvement to reduce blood culture contamination | Collection of specimens |
| Evans (2014) ^128^ | Long Term Care Medicine Conference | To reduce the number of antibiotic starts for a diagnosis of urinary tract infection (UTI) by 25% through use of evidenced based criteria for ordering of urinalysis | Quality improvement before-after study | Nurse practitioners, nursing home direct care givers (RNs, LPNs, CNAs), physicians (n=?) | Skilled nursing facilities (n=4) | USA | AB prescribing UTI | Assessment of clinical status |
| Fabre (2020) ^129^ | Decennial 2020: International Conference on Healthcare Associated Infections | To improve PCN allergy documentation and increase β-lactam use | Before-after study | Patients aged ≥18 years with a PCN allergy Labor & Delivery unit) (n=382, pre-intervention n=305, post-intervention n=77) | Hospital | USA | PCN allergy documentation | Antimicrobial Medication Management |
| Forbes (2019) ^130^ | ID Week | To study the impact of rapid organism identification on the patient management from a quality improvement perspective | Quality improvement study | Bacteremic patients admitted to ICU (n=17) | Hospital | Canada | Blood cultures | Collection of specimens |
| Friedrichs (2012) ^131^ | International journal of medical microbiology. Conference: 64. Jahrestagung Der Deutschen Gesellschaft Fur Hygiene Und Mikrobiologie | Optimization of antibiotic therapy regimes concerning empirical therapy, length of therapy and adherence to local guidelines | Quality improvement study | Phase 1; 238 surgical records  phase 2: 233 surgical records | Hospital | Germany | Antimicrobial stewardship strategy | Antimicrobial Medication Management |
| Garcia-Reyne (2012) ^63^ | European Congress Of Clinical Microbiology And Infectious Diseases (ECCMID) | The aim of this study was to decrease this contamination rate by conducting an education program for nursing staff | Quality improvement study | Nursing staff of emergency room | Hospital | Spain | QI blood culture contamination | Collection of specimens |
| Hansen (2016) ^132^ | DNP | To decrease antibiotic use in the urgent care setting by way of using a “wait and see” prescription plan for patients with viral syndromes, including acute otitis media (AOM), in children and adults, influenza, sore throats and upper respiratory infections (URI). | Quantitative descriptive study | Patients with viral syndrome diagnosis (n=68) | Urgent Care Centre | USA | Decrease AB prescription by educational intervention | Patient communication, education and information |
| Hodgins (2012) ^133^ | Annual Educational Conference And International Meeting Of The Association For Professionals In Infection Control And Epidemiology (APIC) | To reduce blood culture contamination in the emergency department | Quality improvement study | Nursing staff of emergency department | Hospital | USA | Reduction of blood culture contamination | Collection of specimens |
| Hou (2018) ^134^ | American Professionals Infection Prevention (APIC) Annual Conference | Nor formal aim mentioned.  Report on designing nurse-specific tools to increase the pool of champions of antibiotic stewardship (AS). | Quality improvement project | Nurses (hospital A=281, hospital B=93, hospital C=122) | Hospital | USA | Nurse-involvement stewardship | Collection of specimens |
| Hunstad (2010) ^135^ | European Cystic Fibrosis Conference | To make a survey of nursing practice with i.v. antibiotic home treatment for patients with CF with focus on planning, information and education, cooperation and responsibility. | Survey | Nurses working with CF patients (n=90) | Hospital | Denmark  Norway  Sweden | OPAT | Patient communication, education and information |
| Kerr (2016) ^136^ | Hospital Infection Society Annual Conference | To implement a ward-based antimicrobial stewardship programme for nurses highlighting key opportunities for intervention, encouraging ‘antibiotic conversations ‘and acting as effective safety nets. | Quality improvement study | Nurses (n=13) | Hospital | United Kingdom | Stewardship for nurses | Prompting review |
| Kim (2020) ^137^ | Annual Conference Of The Society For Post-Acute And Long-Term Care Medicine | Examines the knowledge retention of the complexities of urinary tract symptoms and the comfort level of nursing staff when communicating with providers. | Quality improvement study | Nursing staff (RN/LPN, n=17) | Long Term Care Facilities (LTCF) | USA | Nurse education UTI | Assessment of clinical status |
| Lo (2014) ^139^ | Clinical Oncology Society Of Australia Annual Scientific Meeting | This pilot is a novel strategy designed to engage the nursing team to lead antimicrobial stewardship by requesting medical staff to outline an antimicrobial plan when antimicrobial agents are initiated | Quality improvement study | Nurses haematology/  oncology unit | Hospital | Australia | Nurse-led intervention (prompting) | Antimicrobial Medication Management  Prompting review |
| Long (2019) ^138^ | The Lancet Global Health. Conference: CUGH 10th Annual Conference | To reduce the time between patient admission and the administration of the first dose of antibiotics and to reduce the frequency of dosing errors by implementing a plan-do-study-act (PDSA) cycle-based quality improvement project | (retrospective chart review) | Baseline: 26 patients NICU period 2: 33 patients NICU period 3: 28 patients NICU | Hospital | Haiti | Timely antibiotic administration | Antimicrobial Medication Management |
| Mahara (2015) ^140^ | Federation Of Infectious Diseases Societies Of Southern Africa Congress | To ensure adherence to stipulated guidelines | Quality improvement study | Assessors, theatre staff, doctors, nursing and pharmacy personnel | Hospital | South Africa | Perioperative AB prophylaxis | Antimicrobial Medication Management |
| Mahdally (2017) ^141^ | Pediatrics. Conference: National Conference On Education (2017) | Is the prospective implementation of this prediction tool in the management of late-preterm and term infants born in a community teaching hospital? Additionally, we will determine if implementation reduces the rate of EOS evaluation and antibiotic use in this setting. | Quality improvement study | Late pre-term and term infants | Hospital | USA | Risk calculator neonatal sepsis | Assessment of clinical status |
| Patamia (2019) ^142^ | AWHONN Convention 2019 - Association Of Women's Health, Obstetric & Neonatal Nurses | To improve this hospital’s antibiotic stewardship through the use of the web-based Kaiser-Permanente early-onset sepsis (EOS) calculator. | Quality improvement study | Newborns (n=not specified) | Hospital | USA | Neonatal sepsis tool | Assessment of clinical status |
| Stephanou (2014) ^143^ | International Conference On Infectious Diseases | Implemented the practice of administering antimicrobials within an hour following prescription through a national antibiotic stewardship pharmacist-driven “hang-time” process improvement protocol. | Pre-post intervention study | ICU pharmacists (39 hospitals), in collaboration with nursing and infection prevention practitioners | Hospital | South Africa | Timely antibiotic administration | Antimicrobial Medication Management |
| Stukus  (2020) ^145^ | American Association Of Allergy Asthma And Immunology Annual Meeting | No formal aim stated (derived: to clarify reported penicillin allergies among children admitted to an infectious disease service at a paediatric tertiary care referral centre | Quality improvement project | Paediatric patient with penicillin allergy (n=192 | Hospital | USA | Penicillin allergy | Antimicrobial Medication Management |
| Subrayen (2014) ^144^ | International conference on infectious diseases | To improve timing of antibiotic administration in a 214-bed private hospital in Gauteng, South Africa | Quality improvement study | Staff members (n=not specified) | Hospital | South Africa | Improvement of timing of antibiotic administration | Antimicrobial Medication Management |
| Taneja  (2014) ^146^ | Annual Conference Of The Association For Professionals In Infection Control And Epidemiology | Reduction of emergency department blood culture contamination rate using Six Sigma methodology | Quality improvement study | Nurses emergency department (n=not specified) | Hospital | USA | Reduction of blood culture contamination | Collection of specimens |
| Travis (2011) ^147^ | Thesis | To determine the effect of a nurse-led educational intervention on urgent care patients' knowledge on viral and bacterial infections and the proper use of interventions | Non-randomized trial | Patients urgent care (n=75) | Not specified | USA | Patient education | Patient communication education and information |
| Vo (2018) ^148^ | ISPOR Europe 2018: New Perspectives For Improving 21st Century Health Systems | To determine the prevalence, potential clinical outcome and factors associated with MEs related to antibiotics during preparation and administration by nurses. | Prospective study | Nurses from 3 clinical wards (n=not defined) | Hospital | VIETNAM | Antibiotic administration | Antimicrobial Medication Management |
| Waisbeck (2013) ^149^ | American Society Of Haematology Annual Meeting | We implemented a nurse-based program to reduce the time from first fever or clinical empiric antibiotics therapy (EAT) indication to EAT infusion. A senior nurse was in charge of implementing the program in the onco-haematological and bone marrow transplantation unit | Quality improvement study | Before : neutropenic patients (n=20) after neutropenic patients (n=17) | Hospital | Brazil | Timely antibiotic administration | Antimicrobial Medication Management |
| Witts(2016) ^150^ | American Professionals Infection Prevention (APIC) Annual Conference | Not formally described prevention of treatment of asymptomatic bacteriuria (ASB) | Quality improvement study | Staff at Long Term Care Facility (LTCF) (prescribers, administrator, director of nursing, nurses) | LTCF | USA | Prevention of treatment of ASB | Assessment of clinical status |
| Yu (2019) ^151^ | ID Week | The aim of this project was to determine ideas, concerns, and gaps in knowledge of bedside nurses at a local hospital level | Survey | Nurses (n=112) | Hospital | USA | Perception and practice of nurse AMS | Collection of Specimen |

# Supplementary file S4: Critical appraisal with Mixed Method Appraisal Tool (MMAT)

**Table MMAT critical appraisal Qualitative studies**

| QUALITATIVE STUDIES | | | | | | | | | | |
| --- | --- | --- | --- | --- | --- | --- | --- | --- | --- | --- |
| Questions | S1 are there clear research questions  S2. do the collected data allow to address the research questions  1.1 I the qualitative approach appropriate to answer the research question?  1.2 are the qualitative data collection adequate to address research question?  1.3 are the finding adequately derived from the data?  1.4 is the interpretation of results sufficiently substantiated by data?  1.5 is there coherence between qualitative data sources, collection, analysis and interpretation? | | | | | | | | | |
| Author (year) |  | S1 | S2 | 1.1 | 1.2 | 1.3 | 1.4 | 1.5 | MMAT-score | Notes |
| Black et al (2019) ^39^ | Health Care Providers' Perceptions of Antimicrobial Use and Stewardship at Acute Care Hospitals in Nova Scotia | Y | Y | Y | Y | Y | Y | Y | 100 | S2. described as "thematic analysis" |
| Broom (2017) ^40^ | Nurses as Antibiotic Brokers: Institutionalized Praxis in the Hospital | Y | Y | Y | Y | Y | Y | Y | 100 |  |
| Broom (2017) ^42^ | Clinical and social barriers to antimicrobial stewardship in pulmonary medicine: A qualitative study | Y | Y | Y | Y | Y | Y | Y | 100 |  |
| Broom (2019) ^43^ | The drivers of antimicrobial use across institutions, stakeholders and economic settings: a paradigm shift is required for effective optimization | Y | Y | Y | Y | Y | Y | Y | 100 | 1.4 & 1.5. quotes in supplemental data |
| Broom (2019) ^41^ | How do hospital respiratory clinicians perceive antimicrobial stewardship (AMS)? A qualitative study highlighting barriers to AMS in respiratory medicine | Y | Y | Y | Y | Y | Y | Y | 100 |  |
| Chaaban (2019) ^49^ | Decisional issues in antibiotic prescribing in French nursing homes: An ethnographic study | Y | Y | Y | Y | Y | Y | Y | 100 |  |
| Currie (2020) ^52^ | Mechanisms affecting the implementation of a national antimicrobial stewardship programme; multi-professional perspectives explained using normalisation process theory | Y | Y | Y | Y | Y | Y | Y | 100 |  |
| Dowson (2020) ^59^ | Antimicrobial stewardship near the end of life in aged care homes | Y | Y | Y | Y | Y | Y | Y | 100 | data collection: semi-structured interviews, of which 2 are not recorded but documented with fieldnotes. Unclear how this accounted for in the analysis |
| Dowson (2020) ^58^ | The role of nurses in antimicrobial stewardship near the end of life in aged-care homes: A qualitative study | Y | Y | Y | Y | Y | Y | Y | 100 | data collection: semi-structured interviews, of which 2 are not recorded but documented with fieldnotes. Unclear how this accounted for in the analysis |
| Gouloupoulos (2019) ^65^ | Attitudes and beliefs of Australian emergency department clinicians on antimicrobial stewardship in the emergency department: A qualitative study | Y | Y | Y | Y | Y | Y | Y | 100 |  |
| Kilpatrick (2019) ^72^ | Antimicrobial stewardship and infection prevention and control in atopic dermatitis in children | Y | Y | Y | CT | CT | CT | CT | 20 | 1.2 ...the sample size might appear limited and could be perceived as a limitation to the study How recruitment is performed is not entirely clear 1.3. small amount of quotes (not covering all emerging themes 1.5. Data-analysis not extensively described |
| Kirby (2020) ^71^ | Reconsidering the nursing role in antimicrobial stewardship: a multisite qualitative interview study | Y | Y | Y | Y | Y | Y | Y | 100 |  |
| Lim (2014) ^73^ | Antimicrobial stewardship in residential aged care facilities: need and readiness assessment | Y | Y | Y | Y | Y | Y | Y | 100 |  |
| Lohfield (2007) ^77^ | Evidence-based clinical pathways to manage urinary tract infections in long-term care facilities: a qualitative case study describing administrator and nursing staff views | Y | Y | Y | Y | Y | Y | Y | 100 | S1: described as results of qualitative study on acceptability of using UTI clinical pathways in LTCFs as a standard protocol. |
| Mula (2019) ^83^ | An exploration of workarounds and their perceived impact on antibiotic stewardship in the adult medical wards of a referral hospital in Malawi: a qualitative study | Y | Y | Y | Y | Y | Y | Y | 100 | S1: aim of study is to explore nurses & doctors workaround S2: 3 focusgroups with doctors, pharmacists & lab technicians. Nurses practices were observed, afterwards interview |
| Ramly (2020) ^88^ | Workflow Barriers and Strategies to Reduce Antibiotic Overuse in Nursing Homes | Y | Y | Y | Y | Y | Y | Y | 100 |  |
| Rout (2017) ^93^ | Exploring the role of the ICU nurse in the antimicrobial stewardship team at a private hospital in KwaZulu-Natal, South Africa. | Y | Y | Y | Y | CT | Y | Y | 80 | 1.2. Although part of coding process shown, not clear if e.g. coding was done independently. Not clear how in detail 'academic rigour" was established. Research familiarity with ICU culture, here presented as means to credibility, can also bring bias into research findings. |
| Rout (2020) ^94^ | Perceived barriers to the development of the antimicrobial stewardship role of the nurse in intensive care: Views of healthcare professionals | Y | Y | Y | Y | CT | CT | Y | 60 | 1.2. Only 6 clinical nurses working on ICU involved, other 2 nurses hospital management, 7 medical profession and 1 pharmacist. Not clear if this is adequate. 1.3. insufficient data available 1.4. insufficient data available |
| Schmitz (2013) ^97^ | Quality of blood culture testing - a survey in intensive care units and microbiological laboratories across four European countries | Y | Y | N | Y | CT | CT | CT | 20 | *additional file with interview guide not available (to determine whether this is, in fact, a qualitative study *data-analysis and results are quantitively presented, not clear why is chosen for a qualitative design instead of e.g. quantitative design (e.g. as in title described) |
| Schouten (2007) ^96^ | Barriers to optimal antibiotic use for community-acquired pneumonia at hospitals: a qualitative study | Y | Y | Y | Y | Y | Y | Y | 100 |  |
| Seshadri (2020) ^98^ | There is no one to pick up the pieces": Sustainability of antibiotic stewardship programs in nursing homes | Y | Y | Y | Y | Y | Y | Y | 100 |  |
| Valmadrid (2021) ^104^ | The impact of health care provider relationships and communication dynamics on urinary tract infection management and antibiotic utilization for long-term care facility residents treated in the emergency department: A qualitative study | Y | Y | Y | Y | Y | Y | Y | 100 |  |
| van Buul (2014) ^105^ | Factors influencing antibiotic prescribing in long-term care facilities: a qualitative in-depth study | Y | Y | Y | y | Y | Y | Y | 100 |  |
| van Gulik (2020) ^106^ | Barriers and facilitators to integrating antimicrobial stewardship into clinical governance and practice: a Thai case study | Y | Y | Y | Y | Y | Y | Y | 100 | 1.3. transcripts translated from Thai to English, then analysed, possible "lost in translation"? No additional data available |
| Walker (2000) ^107^ | Why are antibiotics prescribed for asymptomatic bacteriuria in institutionalized elderly people? A qualitative study of physicians' and nurses' perceptions | Y | Y | Y | Y | CT | Y | Y | 80 | Convenience sampling Analysis of data performed by 1 researcher, no clear description of analytical process |
| Wong (2020) ^110^ | Empowerment of nurses in antibiotic stewardship: a social ecological qualitative analysis | Y | Y | Y | Y | Y | Y | Y | 100 |  |

**Table MMAT critical appraisal Quantative Non-randomized studies (n=33)**

| QUANTATATIVE NON-RANDOMIZED STUDIES | | | | | | | | | |
| --- | --- | --- | --- | --- | --- | --- | --- | --- | --- |
| Questions | S1 are there clear research questions?  S2. do the collected data allow to address the research questions?  3.1 are the participants representative of the target population?  3.2 are the measurements appropriate regarding both the outcome and intervention (or exposure?  3.3 are there complete outcome data?  3.4 are the confounders accounted for in the design and analysis?  3.5 during the study period, is the intervention administered (or exposure occurred) as intended? | | | | | | | | |
| Author (year) | S1 | S2 | 3.1 | 3.2 | 3.3 | 3.4 | 3.5 | MMAT  SCORE | notes |
| Aiken (2013) | CT | CT | Y | Y | CT | N | Y | 60 | S1: aim of quality project described (instead of aim of research)=> to report the experience of developing and implementing a Surgical Antibiotic Prophylaxis policy as an intervention to change healthcare practitioners prescribing behaviour in a Government hospital in Kenya S2: because research question not determined, not possible to answer this question. 3.3. No absolute data about SSI in patients are given, only risk ratio's3.3 all patients who were operated upon were followed up. Total of patients under surveillance (n=3343), not clear if there are any lost-to-follow up (not mentioned) 3.4. No distinction is made between patient characteristics before and after implementation of policy. Analysis adjusted after results were collected and no change was seen. |
| Almaki (2017) | Y | N | CT | Y | Y | CT | CT | 40 | S2: identification of barriers by nurses, only described that nurses were asked to rank 7 possible barriers to administering antibiotics <1 hour, based on their experience 3.1. Patients were retrospectively identified by prescription of vasopressors and antibiotics, thus leaving out those who are possibly not sick enough to have vasopressors (yet) 3.4. Patients in the post-intervention group had higher BMI and APACHE II score.  3.5. Not described (adherence/compliance) |
| Bunsow (2015) | Y | N | N | CT | CT | CT | Y | 20 | S2: to determine the capacity of the HCW in the identification of sepsis=> determined by asking question if HCW thought patient had sepsis, clinical data for sepsis were only collected in intervention group. No further clarification of variable 3.1.only patients who had blood cultures sent from 9 AM-3 PM (Mon-Fr) 3.2. No data on sepsis recognition of HCW, data on satisfaction with interviews with nurses based on general impression of interviewer. 3.3. Non-intervention group no data on sepsis recognition. SA 3.4. see 3.1. Not clear how well randomization based on patient identification number performs (although no statistical difference between groups is presented). Contamination effects of intervention? Not clear what information was shared with nurses, and how this may have influenced treatment. |
| Cooper (2017) | Y | Y | Y | Y | CT | CT | Y | 60 | 3.3 &3.4. Difference in resident census pre- and post-intervention (due to change of resident group matrix). No data on e.g. staffing ratios. Not clear how many residents were under surveillance for UTI pre and postintervention. Knowledge scores: no data on number of participants. |
| Daniels (2018) | Y | Y | CT | CT | CT | CT | CT | 0 | 3.2. no characteristics given, nor comparison in patient characteristics between baseline and post-intervention period 3.2, 3.3., 3.4., and 3.5.: insufficient data to appraise methodogical rigour |
| Davis (2016) | Y | Y | CT | Y | Y | CT | Y | 60 | 3.1 no demographics on patients 3.4 retrospective chart review |
| Dhudasia (2018) | Y | Y | Y | Y | Y | Y | Y | 100 | S1. Quality improvement study; aim was to describe the implementation of a sepsis risk calculator & measure differences in AB use & laboratory testing. |
| Dowson (2019) | Y | Y | CT | Y | Y | CT | Y | 60 | 3.1.no characteristics of participants were given |
| Fabre (2020) | Y | Y | Y | Y | CT | CT | CT | 40 | 3.3. No data available 3.4. no data available (or comparison) on patient characteristics/ward dynamics as in e.g. staffing 3.5. no data available |
| Geerlinks (2020) | Y | Y | Y | Y | CT | CT | CT | 40 | 3.3. comparison with historical cohort, only data compared for confirmed febrile neutropenia |
| Gillespie (2013) | Y | N | Y | Y | Y | CT | CT | 60 | *RQ: to assess influence of nurse education on AB use & clinical practice. Measured outcome: number of IV line days as proxy of AB use? *statistical tests not described |
| Grimes-Holsinger (2002) | Y | Y | Y | Y | Y | CT | CT | 60 | 3.4 no information provided 3.5 no information provided |
| Ha (2019) | Y | Y | CT | CT | CT | CT | CT | 0 | 3.1. no description of patient population 3.2. Intervention not measured in terms of compliance/adherence 3.4. No patient data described (on individual level), not clear is patient characteristics are influencing outcome. 3.5. No data on intervention administration as intended, only frequency of ward rounds |
| Lin (2012) | Y | Y | Y | Y | Y | CT | CT | 60 | 3.4 no information provided 3.5 no information provided |
| Linnebur (2011) | Y | Y | Y | Y | Y | CT | CT | 60 | 3.4. facilities were different in baseline characteristics, not mentioned if this was accounted for in the analysis.  3.5. no description of adherence to intervention described |
| McLaughlin (2016) | Y | Y | Y | Y | Y | CT | Y | 80 | 3.4 no information provided |
| McLaughlin (2017) | Y | Y | Y | Y | Y | CT | CT | 60 | 3.4 no information provided 3.5 no information provided |
| Monsees (2020) | Y | Y | Y | Y | CT | CT | N | 40 | 3.1. Intervention primarily used in hospital B.  3.5. Intervention originally intended as fill-out form, only in hospital C used as a conversation tool (laminated paper attached to nurse's bedside charting system). Use of AET only 26% of the 50 nurses who completed post-survey. |
| Perez (2020) | Y | Y | CT | Y | CT | CT | Y | 40 | 3.1. no description of population (newborns) 3.3. no specific information on completeness 3.4. No description of characteristics patient population before and after intervention. |
| Pittinger (2015) | Y | Y | Y | Y | Y | Y | Y | 100 |  |
| Raybardhan (2020) | CT | Y | CT | CT | Y | CT | CT | 20 | S1: aim of quality project described (instead of aim of research)=> Research question to be addressed could be how effective nurse prompting is (and this research question can be answered with the collected data) 3.1 participants (e.g. nurses) not described 3.2 measurement appropriate, but not described how e.g. prompting is measured  3,4 although some confounders are taken into account (mortality, length of stay, readmissions, multiple organ dysfunction score), however, no further data on patient morbidity or e.g staff are presented.  3.5 no data on intervention compliance |
| Recabal (2020) | Y | Y | Y | Y | Y | Y | CT | 80 | 3.5 no data on intervention compliance |
| Reynolds (2020) | Y | Y | Y | CT | CT | CT | CT | 20 | 3.4. No data available. Data collection included all infants with signs of Serious Bacterial Infection (SBI)and /or antibiotic use, TSO only activated when infants presented with fever (post-intervention). No characteristics comparison between before and after intervention shown.  3.5. no data available/quality improvement project; intervention evolving out of data collected and adjusted |
| Saha (2017) | Y | Y | Y | Y | Y | CT | Y | 80 | 3.4 baseline data with regard to number of prescriptions possibly confounded by inadequate documentation |
| Settelmeyer (2018) | Y | Y | Y | Y | Y | CT | CT | 60 | 3.4 no description of statistical analysis 3.5 no data available |
| Shukla (2020) | Y | Y | CT | Y | CT | CT | N | 20 | 3.1 No description of patients (in relation to antibiotic use 3.3. no information 3.4. no information (possible confounder severity of illness in neonates? No data given on this parameter 3.5. Bundle intervention, several adjustments made |
| Sloan (2020) | Y | Y | CT | Y | Y | CT | N | 40 | 3.1. No description of target population (description of sample present, but certain data missing (e.g. reasons for admission)), convenience sample. 3.4. no data provided, but no account for time effects  3.5. Adherence to AMR intervention 57.4% |
| Sutton (2016) | Y | CT | CT | CT | CT | CT | CT | 0 | S2 Data not described in detail, statistical analysis not applied, therefor not possible to determine if research question can be answered by these results 3.1. no patient characteristics were given 3.2. collected data not specified, characteristics of data not clear (e.g. on nurse-influenced care with regard to AB use, data per patient, not per gift of antibiotic, not clear how overall number is calculated. 3.3. not specified, no overall patient numbers given 3.4. no statistical analysis for significance or adjustment for possible confounders 3.5. no data on intervention compliance |
| Tanner (2020) | Y | CT | Y | CT | CT | CT | Y | 40 | S2 No clear description of variables 3.2. variables not clearly defined or described 3.3 Only outcome percentages given, no exact numbers 3.4. No statistical analyses described |
| Walters (2019) | Y | Y | Y | Y | Y | CT | Y | 80 | 3.4 only minor statistical procedures described |
| Webb (2006) | Y | Y | CT | Y | CT | CT | CT | 20 | 3.1 no characteristics of patients give 3.3 no information provided 3.4 no information provided 3.5 no information provided |
| Zabarsky et al (2008) | CT | y | CT | Y | Y | CT | CT | 40 | *researchQ not stated *no description of LTCF patients *no audit of intervention |
| Zimmerman (2014) | Y | y | Y | Y | Y | y | CT | 80 | 3.5 not clear if intervention is given as intended/not clear which part nurses play in this |

**Supplementary file 4:Table MMAT Critical appraisal Quantative descriptive studies (n=18)**

| QUANTATATIVE DESCRIPTIVE STUDIES | | | | | | | | | |
| --- | --- | --- | --- | --- | --- | --- | --- | --- | --- |
| Questions | S1 are there clear research questions?  S2.do the collected data allow to address the research questions?  4.1 is the sampling strategy relevant to address the research question?  4.2 is the sample representative of the target population?  4.3 are the measurements appropriate?  4.4 is the risk of non-response bias low?  4.5 is the statistical analysis appropriate to answer the research question? | | | | | | | | |
| Author (year) | S1 | S2 | 4.1 | 4.2 | 4.3 | 4.4 | 4.5 | MMAT-score | Notes |
| Abahamye (2016) | Y | N | CT | CT | CT | CT | CT | 0 | S2: audit prescription tool=observation of prescriptions. No interviews or observation of doctors and nurses were performed, so factors which could influence prescribing behaviour were not addressed 4.2. Hundred prescriptions were audited (60 inpatient & 40 outpatient), no description of sampling strategy and/or complete description of population. Factors influencing choice and outcome are not described as such, in part they are discussed in the discussion section |
| Beeber (2021) | Y | Y | Y | CT | Y | CT | Y | 60 | 4.4 data on Non-response not available (self-enrolment from online panel), financial incentive). Convenience sample which was overrepresented of RNs with BSN degrees.  4.5. removal of n=66 based on "out-lier" data, no concrete reason given. |
| Bulabula (2018) | Y | Y | Y | N | Y | N | Y | 60 | 4.2. Research question "nurses in Africa", members of ICAn network invited (n=998), responsrate 17.3%, respondents mainly from South Africa. 4.4. responsrate 17.3% |
| Cadavid (2017) | Y | Y | Y | N | CT | N | CT | 20 | 4.2 Nurse educators, not RN themselves 4.4 Responsrate 34% 4.5 Information not supplied |
| Carter (2019) | Y | CT | CT | CT | CT | CT | CT | 0 | 4.1. no description of sampling strategy or characteristics of the respondents 4.2. no description of respondents’ characteristics given 4.3. no description given 4.5. "descriptive statistics" |
| Cooper (2018) | Y | Y | Y | Y | Y | CT | CT | 60 | 4.4. Data measured over 7 consecutive days (temporary influences?) |
| Dos Santos (2016) | Y | N | CT | CT | N | CT | N | 0 | S2.: aim is to identify risk factors. Results describe medication errors. 4.1. No characteristics described. Possible bias by selection of those charts/patients of which nursing information exists 4.2.no characteristics available 4.3.no clear description of variables 4,4.lack of information 4.5.statistical analysis, based on information provided, less appropriate |
| Fehily (2014) | Y | Y | Y | Y | Y | Y | Y | 100 |  |
| Greendyke (2018) | Y | Y | Y | Y | CT | N | Y | 60 | 4.3. Variable information lacking (supplementary material only shows survey questions, not clear how they were developed) 4.4 Responsrate 13% |
| Hoefel (2004) | Y | Y | Y | CT | Y | CT | Y | 60 | 4.2 no information 4.4. no information |
| Jayaweerasingham (2019) | Y | Y | Y | CT | CT | CT | CT | 20 | 4.2 no information provided on sampling population 4.3 no information questionnaire development provided 4.4. no information provided 4.5 no information provided |
| Le (2006) | Y | Y | Y | Y | Y | N | CT | 60 | 4.4 retrospective cohort/data non-retrievable, change of data collection form 4.5 no information provided |
| Merril (2019) | Y | Y | Y | Y | Y | N | Y | 80 | 4.4. Responsrate 15,8%. No information on different percentages per hospital. |
| Monsees (2018) | Y | Y | Y | Y | Y | N | Y | 80 | 4.4 responsrate 16.4% |
| Musmar (2014) | Y | Y | Y | Y | Y | Y | Y | 100 |  |
| Roberts et al. (2017) | Y | Y | Y | Y | Y | CT | Y | 80 | 4.4. Responsrate 82%. Face-to-face invitation + reminder.  In data analysis section comparison with Chi-square described. However, no such analysis presented. |
| Yeoh (2020) | Y | Y | N | N | Y | N | CT | 20 | 4.1. only patients included who were on antimicrobials (as inpatients) longer than 72 hours 4.2. 48.1% of patients are admitted to geriatric unit, most common antimicrobial oseltamivir. Convenience sampling at the time of an influenza outbreak at the geriatric ward. 4.4. Survey questions not all answered, unclear to what cause. Surveyer-adminstered questionnaire, can result in socially desirable responses.  4.5. Statistical analyses not described. |
| Yogo (2016) | Y | Y | Y | Y | Y | CT | Y | 80 | 4.4 retrospective identification of cases through medical documentation system |

**Table IIID MMAT Critical appraisal of Mixed-Method Studies (n=3)**

| MIXED-METHODS STUDIES | | | | | | | | | |
| --- | --- | --- | --- | --- | --- | --- | --- | --- | --- |
| Questions | S1 are there clear research questions?  S2. do the collected data allow to address the research questions?  5.1 is there an adequate rationale for using a mixed methods design to address the research question?  5.2 are the different components of the study effectively integrated to answer the research question?  5.3 are the outputs of the integration of the qualitative and quantitative components adequately interpreted?  5.4 are divergences and inconsistencies between quantative and qualitative results adequately addressed?  5.5 do the different components of the study adhere to the quality criteria of each tradition of the methods involved? | | | | | | | | |
| Author (year) | S1 | S2 | 5.1 | 5.2 | 5.3 | 5.4 | 5.5 | MMAT-subscore |  |
| Mula, Solomon & Muula (2019) | Y | Y | Y | Y | CT | Y | N | 60 | considering both parts of study, information is missing on data collection and analysis, therefore not always possible to give proper judgment on criteria |
| Broom (2019) | Y | Y | Y | Y | Y | Y | Y | 100 |  |
| Ervin (2021) | Y | Y | N | CT | CT | CT | CT | 0 | 5.5. Qualitative methodology adequately described |

**Supplementary file S4: Visual representation of critical appraisal with MMAT**


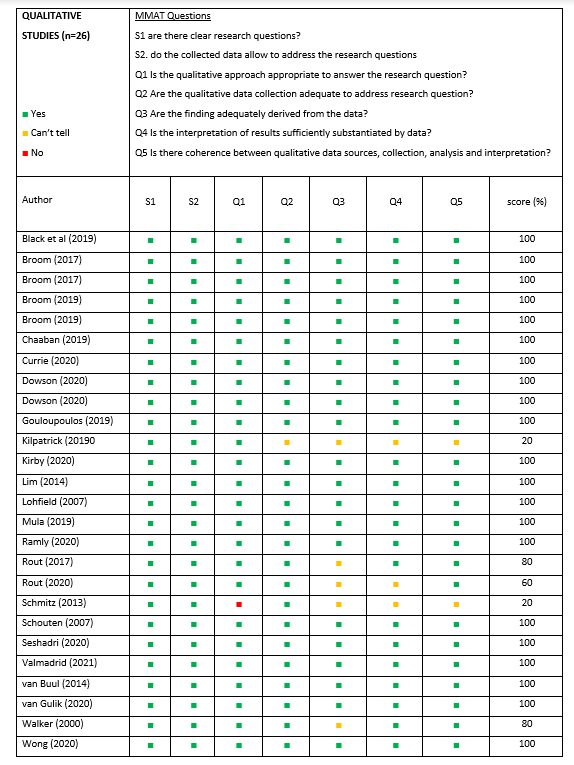


**QUANTITATIVE NON-RANDOMIZED STUDIES (n=33)**


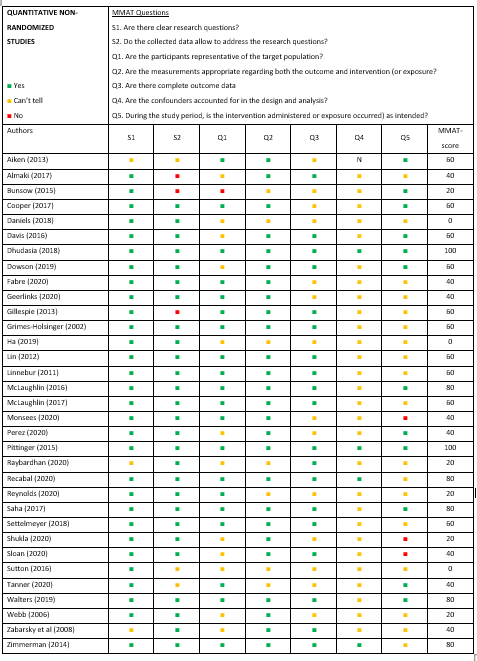


**QUANTITATIVE DESCRIPTIVE STUDIES (n=18)**


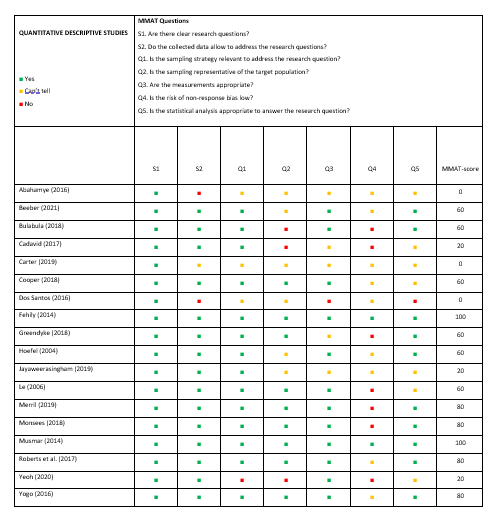


**MIXED METHODS STUDIES**


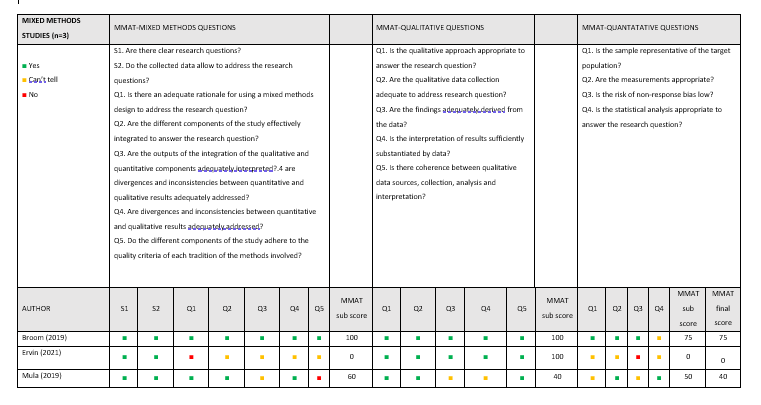


**Supplementary file S4 Figure I MMAT critical appraisal scores**
